# Genome Engineering by RNA-Guided Transposition for *Anabaena* sp. PCC 7120

**DOI:** 10.1021/acssynbio.3c00583

**Published:** 2024-03-06

**Authors:** Sergio Arévalo, Daniel Pérez Rico, Dolores Abarca, Laura W. Dijkhuizen, Cristina Sarasa-Buisan, Peter Lindblad, Enrique Flores, Sandra Nierzwicki-Bauer, Henriette Schluepmann

**Affiliations:** † Biology Department, Utrecht University, Padualaan 8, 3584 CH Utrecht, The Netherlands; ‡ Microbial Chemistry, Department of Chemistry-Ångström Laboratory, Uppsala University, Lägerhyddsvägen 1, 751 20 Uppsala, Sweden; § Instituto de Bioquímica Vegetal y Fotosíntesis, CSIC and Universidad de Sevilla, Avenida Americo Vespucio 49, Sevilla 41092, Spain; ∥ Department of Biological Sciences, Rensselaer Polytechnic Institute, 110 Eighth Street, Troy, New York 12180-3590, United States

**Keywords:** *Anabaena*, CRISPR-associated transposon (CAST), genome engineering, RNA-guided transposition, minion sequencing, *de novo* genome assembly

## Abstract

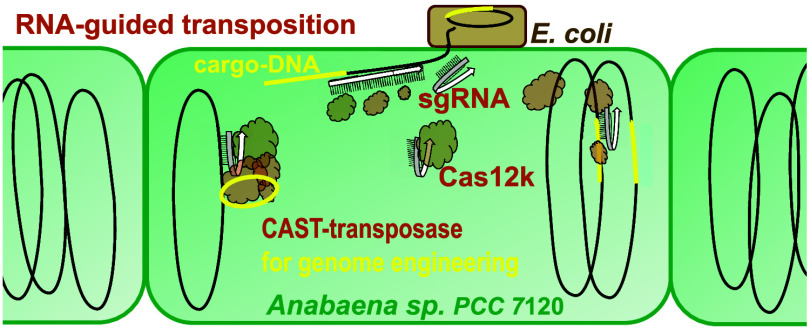

In genome engineering, the integration of incoming DNA has been dependent on enzymes produced by dividing cells, which has been a bottleneck toward increasing DNA insertion frequencies and accuracy. Recently, RNA-guided transposition with CRISPR-associated transposase (CAST) was reported as highly effective and specific in *Escherichia coli*. Here, we developed Golden Gate vectors to test CAST in filamentous cyanobacteria and to show that it is effective in *Anabaena* sp. strain PCC 7120. The comparatively large plasmids containing CAST and the engineered transposon were successfully transferred into *Anabaena* via conjugation using either suicide or replicative plasmids. Single guide (sg) RNA encoding the leading but not the reverse complement strand of the target were effective with the protospacer-associated motif (PAM) sequence included in the sgRNA. In four out of six cases analyzed over two distinct target loci, the insertion site was exactly 63 bases after the PAM. CAST on a replicating plasmid was toxic, which could be used to cure the plasmid. In all six cases analyzed, only the transposon cargo defined by the sequence ranging from left and right elements was inserted at the target loci; therefore, RNA-guided transposition resulted from cut and paste. No endogenous transposons were remobilized by exposure to CAST enzymes. This work is foundational for genome editing by RNA-guided transposition in filamentous cyanobacteria, whether in culture or in complex communities.

## Introduction

Cyanobacteria are of critical importance for the biogeochemical cycling of carbon and nitrogen and therefore substantially influence climate and primary production on earth.^1^ Recent insights highlight the importance of symbioses in these cycles.^2^ Moreover, cyanobacteria are capable of forming complex communities; they form symbioses with eukaryotic organisms including algae, plants, fungi, protozoa, and invertebrate animals such as sponges and ascidians. This is in part because of their versatile secondary metabolism.^3^ Filamentous cyanobacteria from the order Nostocales typically produce heterocysts that can fix dinitrogen in symbioses with plants from all land plant lineages.^4^ Large sequencing data sets are growing in number, expanding our understanding of those associations. However, the understanding of biogeochemical interdependences at the molecular level within these associations has been hampered by the relative genetic intractability of the mostly polyploid cyanobacteria, whether in culture or in symbiotic communities.

Genetic alteration in filamentous cyanobacteria has been accomplished mostly in a few species from the *Nostoc*/*Anabaena* genus complex. DNA cargo transfer into these filamentous cells was achieved by natural competence, electroporation, *Escherichia coli*-mediated conjugation, and *Agrobacterium*-mediated transfer.^5^ Stabilization of the incoming DNA was further achieved not only by methylation of the cargo in donor cells^6^ but also by engineering the DNA sequence such that the DNA may replicate and/or be used as a substrate for homologous recombination. This allowed for the integration of the engineered DNA into target loci on either a plasmid or the chromosome. In all cases, the integration of the cargo DNA was catalyzed by rate-limiting enzymes from the target cell either by homology-directed repair or by end-joining repair. More recently, RNA-guided CRISPR-associated nucleases have been introduced in cyanobacteria to catalyze changes at specific bases more efficiently or cause larger deletions at the target sites of the polyploid species of *Anabaena*, in spite of toxicity issues.^7−9^ However, the RNA-guided nucleases Cas9 or Cpf1 do not permit to track or select with a tag those cells bearing the edit, whether by using a fluorescent or a selection marker. Sequence- and species-directed gene disruption with such a tag is of particular importance to follow genome-edited bacteria in complex mixtures.

The efficient sequence-directed transposition of DNA has been obtained recently in Gram-negative bacteria when catalyzing the insertion of the cargo DNA with CRISPR-associated transposases. Two systems that permit RNA-guided DNA transposition have been studied in *E. coli*: the systems of types I–F and V–K.^10,11^ The type I–F from *Vibrio cholerae* uses a multiprotein effector consisting of the type-characteristic Cas3 protein complexing with CASCADE (complex for antiviral defense) proteins.^10^ The type V–K uses the single effector protein Cas12k, which was discovered in filamentous cyanobacteria, including *Scytonema hofmannii*.^11^ In *E. coli*, CAST increased the frequency of insertion of the donor DNA up to 80% without selection. Importantly, it furthermore afforded its guided insertion because the effector protein binds small RNA that guides the transposition. The mechanism of transposition was not thoroughly investigated but both cut- and copy-paste have been reported.^11^

The CASCADE required more proteins to be expressed than the type V–K, did not transpose the DNA cargo over 1 kbp in size at high efficiency, and reproducibly inserted it 46–55 bases after the PAM, yet in varying orientations.^10^ In contrast, the type V–K from *S. hofmanni* inserted the cargo DNA up to 10 kbp long, 60–66 bp after the PAM in the orientation 5′ left end (LE) to right end (RE) 3′ at high frequency. The ends of the Tn7-derived type V–K cargo transposon consist of the 150 bp LE and the 90 bp RE, encoding 3 and 4 transposase-binding sites, respectively.^11^ The *S. hofmanni* V–K (CAST) system was plagued with significant off-target insertions,^10^ and it may be affected strongly by transcription at the target locus.^12^ Transcripts likely titrate away the guide RNA in a way similar to DNA oligonucleotides or, possibly, the RNA polymerase complex displaces the CAST complex.^13,14^

Type V–K transposition was reconstructed in vitro; it required TnsB known to join the 3′ ends of Tn7 with the target DNA; TnsC, an ATP-dependent transposase activator known to form heptameric rings on DNA; and TniQ known to recruit TnsC to the target DNA.^15−19^ It also needed Cas12k, which, like Cas9, is related to TnpB from the IS605 transposons.^20^ Unlike TnsA, Cas12k did not have the active site known to break the 5′ end of the Tn7 ends; Cas12k required two small RNAs: the constant transactivating RNA that formed a duplex with a part of the otherwise variant crRNA sequence. To specify new CAST targets with ease, the two small RNA binding to Cas12k have been expressed as a single guide RNA (sgRNA) fusion and type two restriction enzyme cutting sites allow seamless cloning of the variable target sequence; the sgRNA design has been validated and optimized.^8^

In order to engineer genomes of filamentous cyanobacteria by efficient RNA-directed transposition, we herewith present the development of synthetic biology vectors with the elements from type V–K CAST comprising the transposase proteins and the cargo transposon designed to study genome editing by way of RNA-guided and catalyzed insertion of the transposon cargo in filamentous cyanobacteria. We tested RNA-guided transposition into highly expressed gene fusions in mutants of *Anabaena* sp. strain PCC 7120 (*Anabaena*), varying the target loci while keeping the sgRNA constant; we further varied the sgRNA sequence, PAM, and strand. We characterized the insertions obtained in transconjugants by PCR, confocal microscopy, and whole genome sequencing/de novo assembly to determine the mechanism of insertion and check for eventual off-target effects. We expect that the described approach will facilitate the targeted inactivation of genes in *Anabaena* and eventually to extend the use of this approach to complex cyanobacterial systems such as symbiotic associations.

## Results

### CASTGATE Elements to Share for RNA-Guided Transposition in Cyanobacteria

**Figure 1:**
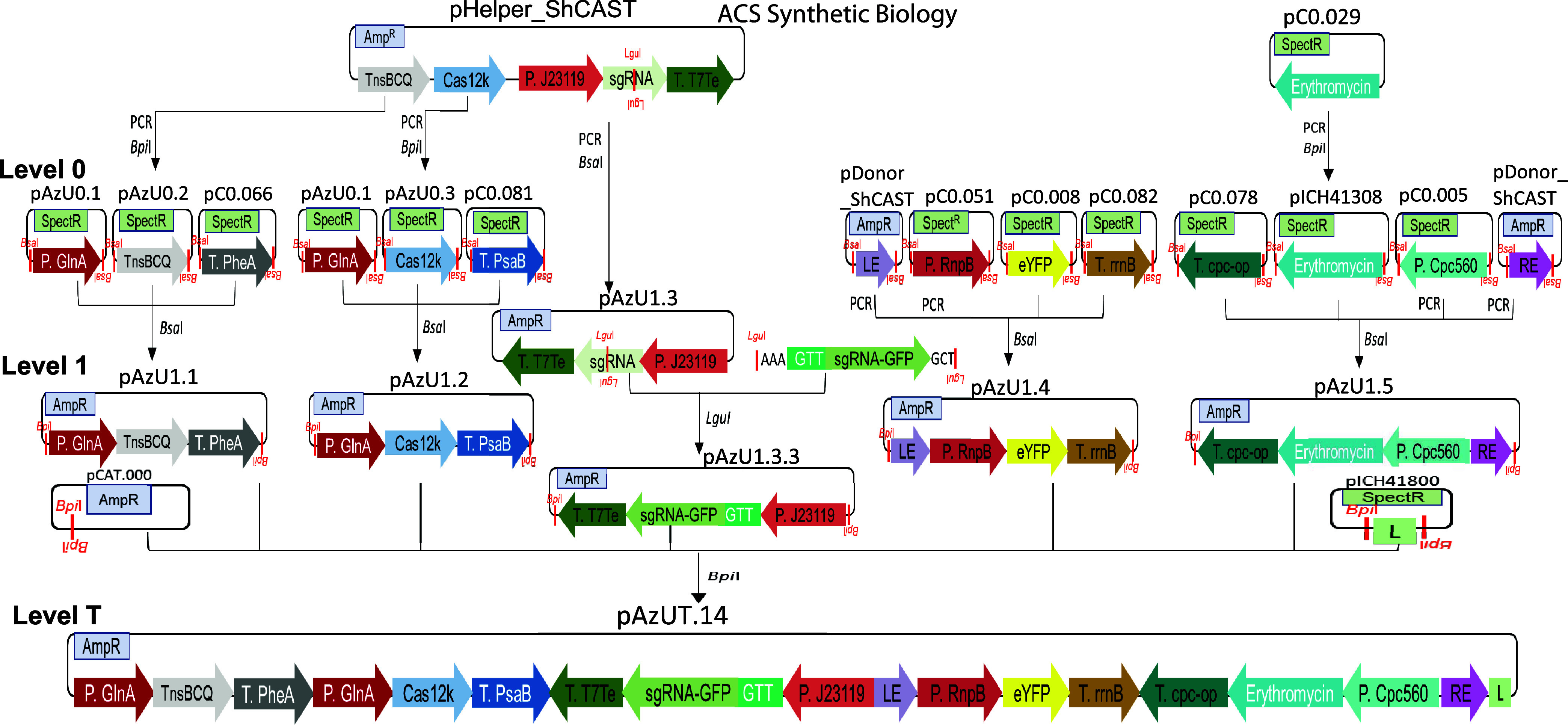
Cloning strategy to obtain the modules and vectors of the CASTGATE. The level 0 plasmids with names beginning with pC0 and the donor for the level T plasmid, pCAT.000, a replicative and conjugative vector, were from the CyanoGate kit.^25^ The level 0 pICH41308 and the level 1 pICH41800 providing the linker (L) at position 6 in the T-level assemblies were from the MoClo kit.^40^ First, sequences were domesticated and transferred in the level 0 vectors: pAzU0.1 for P*_gln A_*, pAzU0.2 for the operon encoding TnsB, TnsC, and TniQ (*tnsBCQ*), and pAzU0.3 for Cas12k, which originated from pHelper_ShCAST.^29^ Alternatively, sequences were PCR-amplified for direct insertion into level 1 vectors, as in the case of the cargo transposon LE and RE, which originated from pDonor_ShCAST^29^ and the expression cassette of the sgRNA scaffold from pHelper_ShCAST that allows to ligate target-specific sequences in LguI restriction sites. Second, expression cassettes were assembled in level 1 for the specific positions (1.1–1.5) of the level T assemblies: pAzU1.1, low nitrogen-inducible expression of *tnsBCQ*; pAzU1.2, low nitrogen-inducible expression of Cas12k; pAzU1.3, the sgRNA scaffold, which, when the GFP-specific target sequence (AAAGTT-GFPgRNA-GCT*) was ligated in the LguI site, yielded pAz1.3.3; pAzU1.4, with at the 5′ LE, then the cassette for constitutive expression of eYFP; pAzU1.5, with at the 5′ the cassette for constitutive erythromycin resistance followed at the 3′ with the RE. Third, the level 1 plasmids were combined to generate level T pAzUT.14, encoding the CAST machinery followed by the cargo DNA flanked by the LE and RE. CAST component level 1 plasmids were replaced with linkers during the final level T plasmid assemblies to test their individual toxicity and efficacy. * three different target sequences within the *gfp* sequence were tested as described in Supporting Figure S2.

CAST elements were toxic when *E. coli* containing vectors encoding them were left on plates for over 1 week. To test the toxicity and efficacy of combinations of CAST elements in cyanobacteria, they were transferred into the Golden Gate cloning vectors collectively called CASTGATE. This approach allows (i) sharing of the individual elements in other synthetic biology studies (Supporting Table S1) and (ii) assembling large vectors systematically with expression cassettes of CAST transposase proteins and the transposon cargo defined by the LE and RE (Figure 1).

Level 0 vectors were generated for sharing individual components modified so as to no longer contain restriction sites for the type IIS restriction enzymes *BsaI* and *BpiI.* The components included the operon *tnsB, tnsC*, and *tniQ*, *cas12k*, and the LE and RE (Figure 1, Level 0). They further contained the *gln A* promoter from *Anabaena* (Pgln *A*; Valladares et al., 2004) (Figure 1, *gln A*) chosen for cyanobacterial-specific CAST protein expression and for the possibility to increase expression when cyanobacteria are grown without nitrogen in the culture medium (Figure 1). The level 1 vector for sgRNA expression with its very strong promoter was designed to transcribe away from the LE because RNA polymerase may interfere with transposase binding;^13,14^ it contained the sgRNA scaffold with LguI restriction sites allowing for the insertion of annealed primers specifying the sequence targeted by the sgRNA (Figure 1, level 1, position 3). The level T plasmids allowed for the final assemblies of CAST elements and engineered transposons; they were generated on backbones of conjugative and replicative vectors, nonconjugative replicative vectors for different methods of transformations, and conjugative but not replicative (suicide) vectors for rapid removal (Figure 1, level T).

The CAST machinery expressed in *E. coli* is toxic (Strecker et al. 2019) yet the CASTGATE vectors listed in Supporting Table S1 where CAST is expressed with P_*gln A*_ proved stable in *E. coli*. We thus attempted transfer of the conjugative replicative vectors listed in Supporting Table S2 into *Anabaena* by triparental conjugation and subsequent antibiotic selection.

### RNA-Guided Transposition Efficiently Targets Expressed Genes Irrespective of the Locus Position in the *Anabaena* Chromosome

Reference vectors containing only the cargo DNA with expression cassettes for cytosolic eYFP (YFP) and erythromycin or spectinomycin/streptomycin resistance (Supporting Table S1, pAzUT.3 and. 4) yielded clones in all of the *Anabaena* strains tested: the wild-type PCC 7120, the derived strains containing the GFP fused to the ammonium transporter protein Amt1 (*amt1::gfp*, CSVT15; (Merino-Puerto et al., 2010)) or the septal protein SepJ (*sepJ::gfp*, CSAM137; (Flores et al., 2007)).

On nitrogen-rich BG11 medium, the following vectors yielded transconjugants: vectors containing all of the CAST and the cargo DNA but the inactive sgRNA, those containing the sgRNA in isolation, or those with all of the CAST elements and the active sgRNA targeting the GFP sequence. Expression of the CAST components, therefore, was well enough repressed behind the P_*gln A*_ on a nitrogen-rich medium, or the inherent toxicity of the CAST proteins was low enough for the transconjugant plasmids to be maintained in *Anabaena*.

On BG11_0_ medium without nitrogen, strain CSAM137 survived the short periods of induction for P_*gln A*_*-*dependent CAST protein expression but not the long periods. In contrast, strain CSVT15 grew on BG11_0_ which allowed to test the toxicity of CAST proteins when P_*gln A*_ strongly drives their expression (Supporting Figure S1). On BG11_0_, Cas12k caused growth inhibition when expressed in isolation (Supporting Figure S1 28 days, pAzUT7) but when expressed with all of the CAST components in transconjugants with pAzUT9, it did not affect the growth.

Homogeneous clones cured of the donor plasmids were obtained at high frequency after sonication and growth on BG11 medium under erythromycin selection when using pAzUT14 in either the CSVT15 or CSAM137 strains. pAzUT14 expressed the sgRNA1 targeting the sense strand of the GFP 163 bp into the 563 bp protein (Supporting Figure S2).

**Figure 2:**
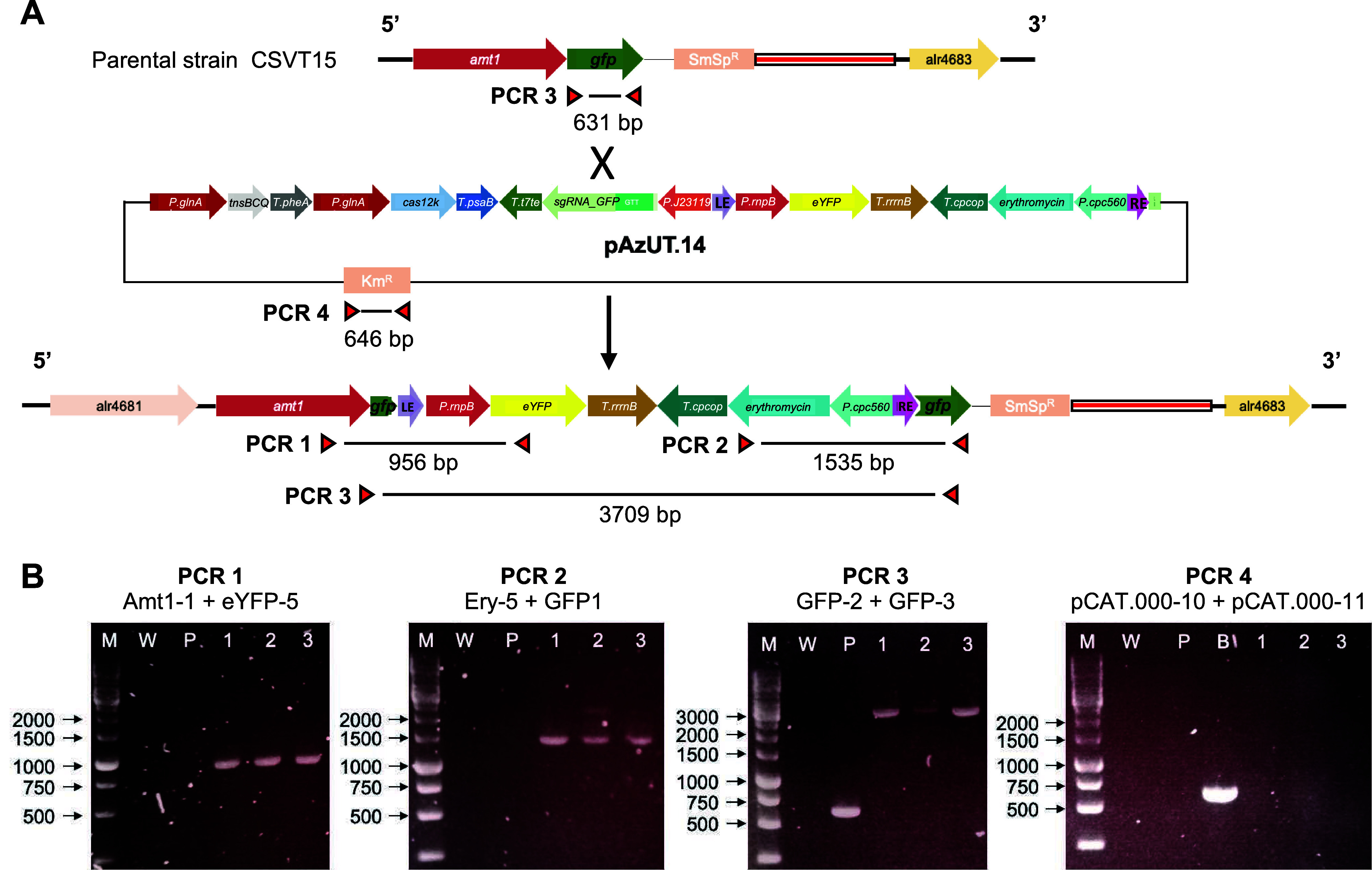
Scheme of the RNA-guided transposon insertion in the *gfp* of the *amt1::gfp* locus of the parental strain CSVT15 and its detection by PCR. (A) Scheme of the *amt1::gfp* locus from strain CSVT15 that, after conjugation and selection on erythromycin, also contained pAzUT.14. pAzUT.14 encoded the RE- and LE- flanked transposon cargo, which would be mobilized by the separately encoded CAST enzymes and sgRNA targeting the *gfp*. When mobilized with complete resolution of the transposase complex, the cargo transposon inserts inside the *gfp* of the *amt1:gfp* fusion. (B) PCR detection of cargo insertion and full segregation in the different exconjugant clones. PCR 1 detects the eYFP integration; PCR 2 detects erythromycin-resistance gene integration; PCR 3 detects *gfp* and thus segregation of the transposed genotype; PCR 4 detects whether the exconjugant had been cured of pAzUT.14. The letters and numbers correspond with (M) 1 kb DNA ladder; (W) *Anabaena* sp. PCC 7120; (P) CSVT15; (B) pAzUT.14; (1) AzUU1 clone 1; (2) AzUU1 clone 4; and (3) AzUU1 clone 8. *Anabaena* sp. PCC 7120 was used as the negative control in all PCR tests. In PCR 3, the CSVT15 strain was used as the positive control.

**Figure 3:**
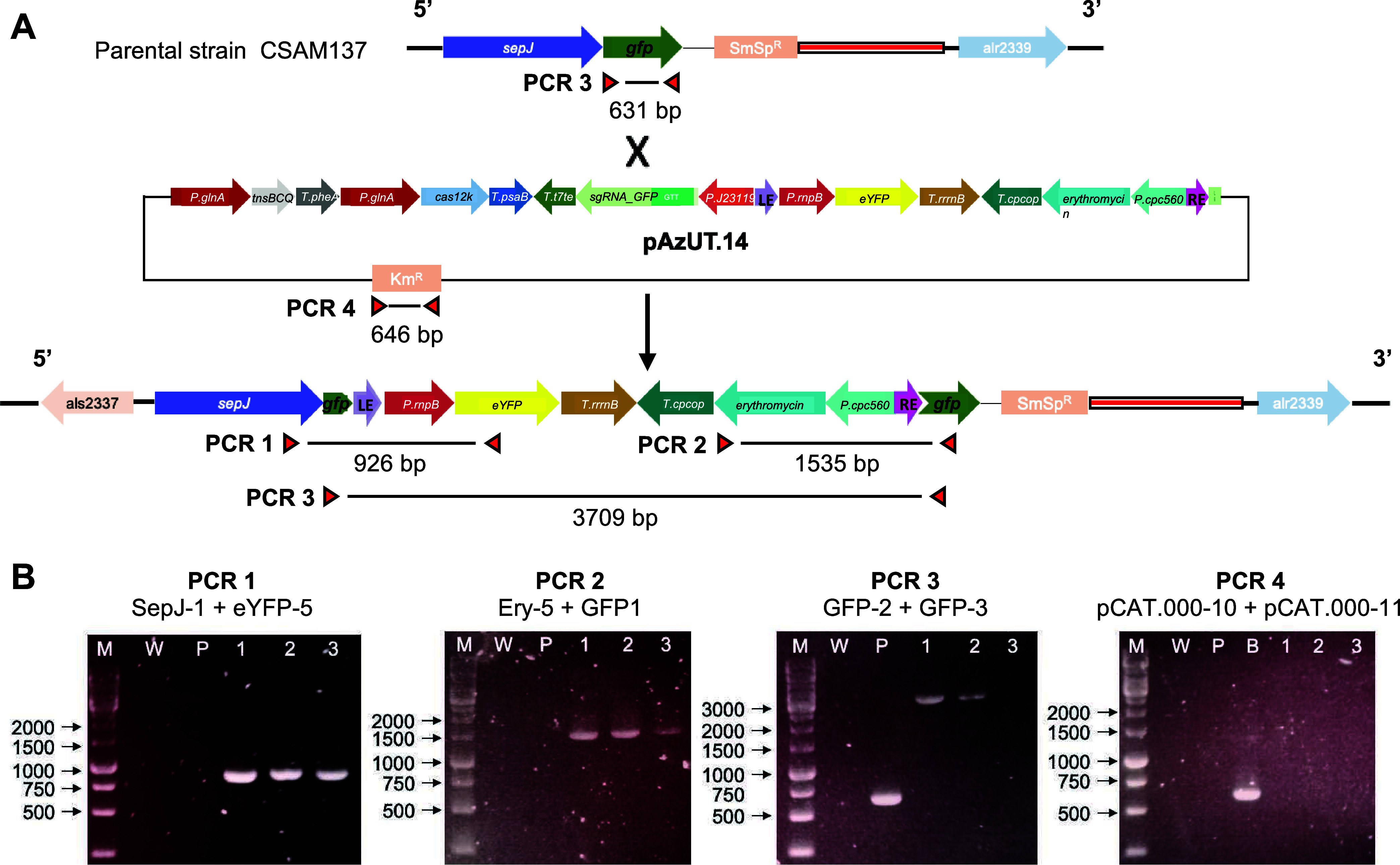
Scheme of RNA-guided transposon insertion in the *gfp* of the *sepJ::gfp* locus of the parental strain CSAM137 and its detection by PCR. (A) Scheme of the *sepJ::gfp* locus from strain CSAM137 that, after conjugation and selection on erythromycin, also contained pAzUT.14. pAzUT.14 encoded the RE- and LE-flanked transposon cargo, which would be mobilized by the CAST enzymes and sgRNA targeting the *gfp*, also encoded in pAzUT.14. When mobilized with resolution of the transposase complex, the cargo transposon inserts inside the *gfp* of the *sepJ:gfp* fusion. (B) PCR detection of cargo insertion and full segregation in the different exconjugants. PCR 1 detects eYFP integration; PCR 2 detects erythromycin-resistance gene integration; PCR 3 detects *gfp* and thus segregation of the transposed genotype; PCR 4 detects whether the exconjugant had been cured of pAzUT.14. The letters and numbers correspond with (M) 1-kb DNA ladder; (W) *Anabaena* sp. PCC 7120; (P) CSAM137; (B) pAzUT.14; (1) AzUU2 clone 3; (2) AzUU2 clone 8; and (3) AzUU2 clone 7. *Anabaena* sp. PCC 7120 was used as the negative control in all PCR tests. In PCR 3, the CSAM137 strain was used as the positive control.

Genotyping of the clones obtained at the *amt1::gfp* locus of CSVT15 was caried out by PCR amplification spanning either ends of the transposon (Figure 2A, PCR 1, PCR 2), the region targeted by the sgRNA (PCR 3) and the pAzUT14 backbone (PCR 4). In all three clones tested, the expected size of the fragments was amplified at either ends and the total size of the inserted transposons was consistent with a single 5′ LE to RE 3′ transposon insertion (Figure 2B, PCR 1, 2, 3). The backbone of the donor vector could not be detected, confirming that the clones were cured of it (Figure 2B, PCR 4). Genotyping the clones obtained at the *sepJ::gfp* locus after curing pAzUT14 from transconjugants of CSAM137 gave a similar result (Figure 3A): amplicons spanning either ends (Figure 3B, PCR 1, PCR 2), amplicons spanning the entire length of the insertion (Figure 3B, PCR 3), and absence of the backbone (Figure 3B, PCR 4). Therefore, PCR genotyping demonstrated that CAST was able to accurately guide the cargo transposon insertion into the highly expressed recombinant GFP, independent of the *Anabaena* locus chosen.

Using the vector pAzUT18, which encodes an sgRNA targeting the sense strand at the wild-type locus *alr3727*, all transconjugant colonies tested by PCR, as described in Supporting Figure S3A, had traces of the cargo transposon insertion (Supporting Figure S3B), unlike the parental wild-type strain (Figure 3B, WT), which confirmed the efficacy of CAST on a wild-type gene.

### Integration of the Cargo DNA Inactivated the Targeted GFP Fusions

**Figure 4:**
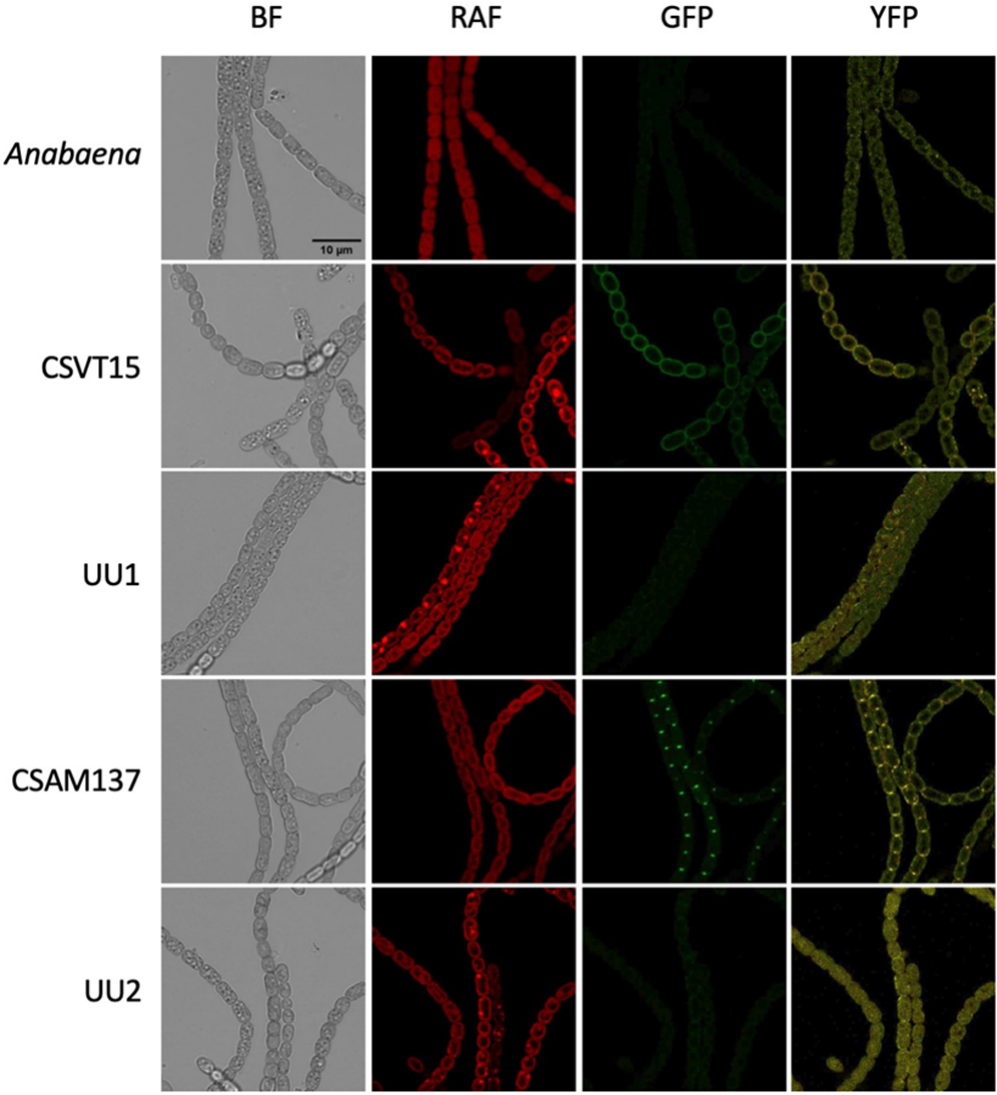
Fluorescence detection of the GFP fusions and the transposon-encoded YFP in *Anabaena* strains. The strains UU1 and UU2 were fully segregated for the transposon insertion and cured of pAzUT.14. Brightness and contrast settings were equal for all image detection types: the bright field (BF) and the fluorescence settings to detect red autofluorescence (RAF), green fluorescent protein (GFP), and yellow fluorescent protein (YFP). *Anabaena*, wild-type strain; CSVT15, the parental strain with the *amt1::gfp* locus; UU1, a CSVT15-derived exconjugant, as shown in Figure 2; CSAM137, the parental strain with the *sepJ::gfp* locus; and UU2, a CSAM137-derived exconjugant, as shown in Figure 3. Scale bar, 10 μm. The images are representative of at least 20 micrographs for each strain gathered from two or three independent microscopy analyses.

Confocal viewing was not ideal for screening because it was not able to distinguish whether the fluorescence detected from the expression of YFP was encoded in the plasmid or the DNA cargo transposon insertion in a chromosomal locus. In strains cured of the plasmids, however, it verified the absence of the GFP fusion protein (Figure 4 GFP) and strong YFP expression in the transconjugants obtained. YFP was seen as yellow fluorescence in the cytosol of all cells of the clones UU1 and UU2 (Figure 4 YFP) compared to the respective parents CSVT15 and CSAM137, which expressed the GFP fusions. This confirmed that the insertion of the RNA-guided transposon cargo into the chromosome loci may be used to tag *Anabaena*, which is known to be polyploid (average number of chromosome 8.2^24^).

We conclude, therefore, that the RNA-guided transposition in *Anabaena* may serve to generate tagged inactivation at expressed loci specified by the sgRNA.

### sgRNA Targeting the Sense Strand of the Expressed GFP and Containing the GTT PAM Was Effective

We next explored how the sgRNA sequences affect the specificity and efficacy of RNA-guided transposition (Supporting Figure S2). The sgRNA from pAzUT14 efficiently targeted the sense strand of the expressed *gfp* fusions and contained in its 5 prime the PAM GTT 162 bases into the *gfp* sequence (Figures 2 and 3). Including the PAM in the sgRNA sequence for strong binding of the sgRNA to Cas12k was attempted because the sgRNA locus on the donor plasmid is protected by the proximity of the LE of the transposon from DNA cargo insertions and thus inactivation. Insertions of the cargo transposon were observed when the sgRNA was used from pAzUT10 that contained the PAM GGTT and targeted the sense strand of *gfp* 271 bp into the coding sequence of the *gfp*, but these were recovered at a much lower frequency (Supporting Figure S2, and data not shown). No insertions were observed for pAzUT12 where the sgRNA contained the PAM GGTT and targeted the antisense strand of *gfp* 563 bp into the *gfp* sequence (Supporting Figure S2, and data not shown).

### Cargo Transposon Inserted Mostly 63 Bases after the PAM and Led to 2–5 Base Duplications at the Insertion Sites

**Figure 5:**
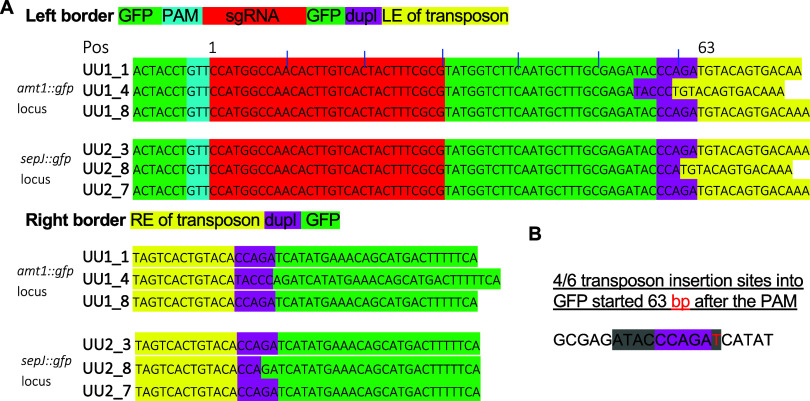
Sequences at the insertion sites of the cargo transposon after RNA-guided transposition into the *gfp* of the *amt1::gfp* or *sepJ::gfp* loci. (A) Sequences that encode the GFP (green) or the LE and RE of the cargo transposon (yellow) are highlighted. In addition, sequences of the *gfp* that were a part of the sgRNA encoded by pAzUT.14 are highlighted that encode the PAM (blue) and the remainder sequence specific part of the sgRNA (red). Resolution of the transposase complex leads to the insertion of nucleotides, causing the small insertions highlighted in purple. The position (Pos) of the insertions was counted starting from the first base after the PAM. (B) The insertion was exactly at position 63 for four of the six independently recovered clones derived from either parent. T in red denotes the LE start base. Similarly, resolution of the transposase led in four of the six clones to a five-base repeat, CCAGA in purple.

PCR results suggested that, in all cases, the cargo transposon was inserted in the 5′ LE to RE 3′ orientation, but PCR could not distinguish whether the insertions had been a result of cut- or copy-paste mechanisms. For accurate sequence information on the integration loci, DNA was extracted from the six independently obtained clones exhibiting RNA-guided transposition (Figures 3 and 4), their respective parental strains, and from the wild-type *Anabaena*. The DNA was then sequenced using a Nanopore flowcell (MinION) collecting at least 50× sequencing coverage for each strain; the reads obtained from sequencing were long and accurate enough for assembly into a single full-length chromosome with a highly accurate sequence for each strain. The sequences immediately adjacent to the cargo transposon were extracted and aligned for comparison (Figure 5). In four of the six genomes analyzed, the LE inserted exactly 63 bases behind the PAM. When comparing sequences at the LE with RE, duplications of the bases at the vicinity of the insertion due to the resolution of the transposase complex were two to four bases long, and in five out of six cases, they were exactly five bases (Figure 5, duplications highlighted in purple). RNA-guided cargo transposon insertions were therefore very reproducible.

### RNA-Guided Transposition Was Unidirectional 5′ LE to RE 3′ and Single-Copy Insertion without the Cointegration of the Donor Plasmid

**Figure 6:**
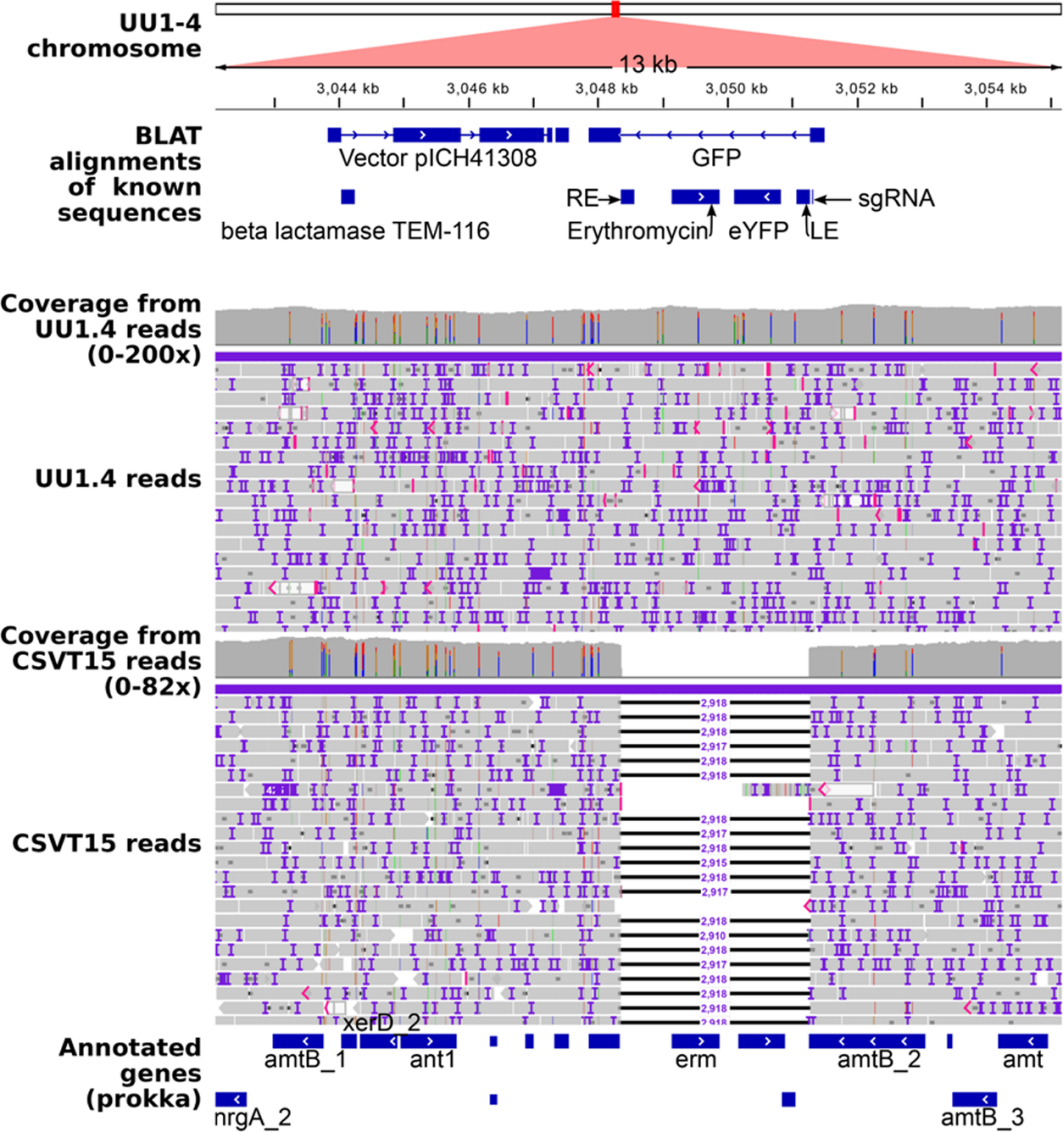
*amt1:gfp* locus in the strain UU1.4 compared to the parental strain CSVT15. Strains were sequenced using MinIon with minimally 50 times coverage and their genomes assembled de novo. The assembly was automatically annotated with Prokka, and known sequences were aligned using the BLAT aligner to the *amt1::gfp* locus. In addition, nanopore (MinION) sequencing reads obtained from the sequencing strain UU1.4 and the parental strain CSV15 were aligned to the assembled genome of UU1.4. The assembly, automated and manual annotation, and the alignments were then visualized in Integral Genome Viewer (IGV) in the 13 kbp region spanning the 2918 bp cargo transposon and the single crossover homologous recombination that yielded the *amt1::gfp* fusion in the parental strain CSVT15. The latter contained vector sequences 3 prime of the *gfp* (Vector pICH41308).

To inspect the overall structure of the loci targeted by the cargo transposon in each clone sequenced, 13 kb regions of the consensus assembly sequences spanning the insertions were viewed in IGV along with aligned reads from the clone and its parent. A typical result is shown for the clone UU1.4 in Figure 6. Alignment of reads obtained from the parental strain CSVT15 revealed some single nucleotide polymorphisms between the clone and the reference strain, yet the foremost difference was the 2918 bp insertion in the clone UU1.4 corresponding in size with the cargo transposon. Automatic annotation (Figure 6, Annotation (prokka)) identified *amt1* as *amtB* based on its homology to *amtB* from *E. coli*. Individual alignments using known sequences (Figure 6, BLAT alignments of known sequences) identified the start of the GFP, the gap caused by the insertion (line with arrows), and the remainder of *gfp*; it furthermore identified the *yfp* (VECTOR_GFPLIKE) and erythromycin resistance. Additionally, downstream of the cargo transposon insertion, it identified the vector sequences used to generate the *amt1::gfp* fusion in the parental strain CSVT15; these vector sequences were inserted through a single crossover homologous recombination event. Results obtained from the analyses of the other six clones investigated revealed an identical mechanism of insertion of the RNA-guided cargo transposon. From the restricted analyses carried out here, we conclude, therefore, that cargo transposon insertions guided by CAST were unidirectional from 5′ LE to RE 3′ with a precise resolution of the 5′ and 3′ ends with cut.

### No Sign of Off-Target Insertions or Remobilization of Endogenous Mobile Elements

We next examined whether exposure to the CAST machinery led to the remobilization of mobile genetic elements (MGEs) already present within the genome of the parental strains.

The automatic detection of indels and recombination events in genomes of the transconjugants and their respective parental strain returned, in the case of the *amt1::gfp* locus, for example, 22 putative events (Supporting Table S3). Manual inspection of the regions corresponding to these events using evidence from the aligned long reads, however, could not verify any changes due to transposon remobilization in the nine events identified in the chromosome (Supporting Figures S6–S11). One deletion from contig_1_2444322_Sniffles2_DEL_5M4 was already present in the parental strain CSVT15 and segregated with more of less penetrance in the clones UU1.1, UU1.4, and UU1.8 (Supporting Figure S6). The events in the plasmids were mostly associated with two transposases each encoded on contig_4 and 5, respectively, the indels did not have clear borders and were difficult to evaluate as we suspected inaccuracies in the plasmid assemblies in repetitive sequences (Supporting Figure S12). We therefore conclude that exposure to the CAST machinery did not cause the remobilization of endogenous mobile genetic elements in the six cases that were analyzed in this study.

## Discussion

### Golden Gate Toolbox Extension for Genome Engineering by RNA-Guided Transposition in Filamentous Cyanobacteria

The discovery of type II restriction enzymes with three- to four-base overhangs opened the way to seamless cloning of many fragments simultaneously. Yet, the most important advance toward synthetic biology was their use in a cloning system with a controlled “vocabulary” in which overhang sequences specify the position and orientation in an expression cassette or a cloning assembly of multiple cassettes or noncoding elements; this allowed sharing of the individual elements in plasmids between laboratories and new combinations to be generated at great speed. Here, we used the vocabulary from the “Golden Gate” system expanding on an existing set of vectors named CyanoGate.^25^ We added a conjugative suicide vector, all of the CAST elements, and the P_gln *A*_ promoter for nitrogen-regulated expression in cyanobacteria under nitrogen starvation.

We combined CAST elements and the cargo DNA in a single conjugative vector (Figure 1), but unlike in ref (26), we inserted the sgRNA cassette in the reverse complement orientation such that RNA polymerase on the strongly expressed sgRNA gene would not affect the transposase binding to the LE. In addition, we designed 34 nt-long sgRNA spacers, well over the minimum required for specific targeting of RNA-guided transposition in vitro.^27^ Also, the PAM is not cleaved by CAST as in the case of Cas9^28^ and insertions near the PAM of the sgRNA would be suppressed by the LE and RE borders of the donor plasmid.^29^ We therefore included the PAM in the sgRNA spacer to test whether the binding of the sgRNA PAM sequence to the N-terminal groove of Cas12k would lead to tighter binding and therefore repression of random transposition.^27^ As presented in the results, this configuration resulted in the specific RNA-guided integration at either of the loci targeted by the sgRNA in *Anabaena* (Figures 2 and 3).

### Accurate Targeting of the RNA-Guided Transposon Cargo in *Anabaena*

In all six transconjugant *Anabaena* clones analyzed in depth, no off-target effects were detected (Supporting Table S3). These effects could have been off-target insertions of the cargo DNA or deletions and remobilization of endogenous mobile elements inside the *Anabaena* genome. Absence of off-target effects contrasted with previous reports on poor target specificity in a variety of Gram-negative bacteria.^26,29^ Results from Xiao et al. (2021) showed that when Cas12 lacks the sgRNA, RNA transposition occurs at a high rate and in random locations; therefore, the authors suggested that sgRNA binding suppresses the random transposition. In our system, the sgRNA was thus expressed at a sufficiently high level such that when bound to Cas12k, it suppressed random transposon insertions.

It is recognized that a higher throughput analysis will be required to investigate off-target effects of the engineered CAST system used here for *Anabaena*. We observed poor growth of clones on BG11_0_ medium, when the P_gln *A*_ directs optimum expression of Cas12k and an sgRNA scaffold, suggesting that the constitutive expression of these CAST components may be toxic to the *Anabaena* cells (Supporting Figure S1). We observed toxicity of the CAST system in *E. coli* (results not shown). The relative growth handicap imparted by the plasmids encoding CAST in BG11_0_ medium was exploited to cure the replicative plasmid containing CAST away from inside the clones exhibiting transposon integration (Figures 2 and 3).

We used the unmodified LE sequence, which is suspected to direct homing via delocalized CRISPR RNA to the tRNA-leu in *S. hofmanii* (sh); it contains a 17-bp motif matching the *sh*tRNA-Leu gene. However, in our construct, the CRISPR direct repeat found upstream of the 17-bp motif in the *sh*CAST locus is missing.^30^ A more thorough understanding of the LE sequence is critically needed in many respects, but most importantly for the design of intron boundaries in the LE and the cargo DNA so as to engineer protein fusions in coding loci targeted by RNA-guided transposition. TnsB-binding sites are the characteristic features of the LE and RE and TnsB was found to bind the backbone of the DNA only and to have a not very strict DNA sequence preference upon binding.^31^

### RNA-Guided Transposition Was Unidirectional from the LE to RE, Reflected a Copy-and-Paste Mechanism, and Was Likely Influenced by RNA

Unidirectional insertion of the cargo (Figure 5) is consistent with the unidirectional formation of the polymeric TnsC complex with the target DNA and Cas12k serving to recruit the TnsB transposase at the target site.^15,19^

Cointegration of the plasmid sequences supplied along with the transposon cargo was reported to occur at frequencies ranging from some 85–19.4%.^32−34^ It was 0.6% in the specific case of the Cas12k-homing endonuclease fusion.^34^ An explanation for cointegration was also proposed: CAST lacks TnsA required to excise the transposon and Cas12k lacks the endonuclease activity to substitute for TnsA. In *Anabaena*, we recovered only single copies of the cargo transposon and there were no plasmid sequence cointegrates (Figure 6). This may be due to the presence of accessory proteins in the cyanobacteria in which Cas12k has evolved to be devoid of the nickase activity typically found in TnsA (and ref (27) therein). Our results are of insufficient throughput to conclude definitely.

The sgRNA targeting the antisense strand of the *gfp* gene fusions at either loci was not effective. This may have been due to the sense transcript RNA duplexing efficiently with the sgRNA and consequently less efficient RNA/DNA heteroduplexing at the target location. For example, the gRNA depletion with DNA oligonucleotides was reported to be effective in the case of Cas9-bound gRNA.^13^ The lesser efficacy of the sgRNA with a spacer containing the GTT PAM may stem from a suboptimal fit at T287 from the WED domain and R421 from the PI domain.^27^

### RNA-Guided Transposition to Understand Genetic Features in Complex Communities Such As Symbioses

RNA-guided transposition may be used to target a locus in a specific organism in a mixture effectively; this has been recently demonstrated in bacterial communities of various complexities.^26^ The advantage of this approach does not only reside in the catalysis, and therefore the efficiency with which the cargo DNA is inserted into the DNA, but also in the insertion of tags at precise locations resulting in engineered loci that may be selected or followed visually in complex mixtures. We used YFP to trace whether the colonies were homogeneous genetically after sonication and prolonged selection (Figure 4). The method lends itself to study the fitness of the cells with engineered loci in mixed communities so as to study the role of the original versus engineered loci in microbe interactions.

We used a replicative plasmid in this initial study to be assured of sufficient expression of the CAST elements and sufficient cargo substrate for transposition. A replicative plasmid is not desired for the precise engineering of loci in microbes within complex communities because this may lead to a larger proportion of off-target insertions of the cargo. Because curing the replicative plasmid used for the delivery of CAST and the cargo is cumbersome, we tested a conjugative suicide vector (pAzUT.17) for delivery of the cargo DNA in a shortened protocol. After consecutive conjugation and sonication (to reduce the length of the filaments), cells were immediately transferred onto BG11_0_ medium for 2 days, and then on BG11 medium and selection. RNA-guided cargo DNA transposition events from conjugation with suicide plasmids were detected in clones that had no trace of the suicide plasmid (Supporting Figure S4).

We conclude that filamentous cyanobacteria, such as *Anabaena*, are amenable to genome engineering using RNA-guided transposition with the Cas12k-based CAST system. We next will need to test the approach in other species and use alternative methods of DNA transfer. We urgently will test the method on symbiotic species, such as *Nostoc punctiforme* and *Nostoc azollae*, especially when present in complex microbial consortia.

## Materials and Methods

### Bacterial Strains and Growth Conditions

*Anabaena* sp. (also known as *Nostoc* sp.) strain PCC 7120 and mutants CSVT15^22^ and CSAM137^23^ were cultured photoautrophically in BG11 or BG11_0_ (without NaNO_3_) media^35^ at 30 °C, under constant white light (35 μE·m^–2^·s^–1^) and shaking. For solid cultures, 1% (w/v) agar (Bacto-Agar, Difco) was added. When required, media were supplemented with 5 μg/mL (solid medium) or 2.5 μg/mL (liquid medium) streptomycin sulfate (Sm), spectinomycin (Sp), or erythromycin (Em).

*E. coli* DH5α (Invitrogen) was used for cloning techniques, while strains HB101 and ED8634, which contain plasmids pRL623 and pRL443,^6^ respectively, were used for conjugation as described in ref (36). All *E. coli* strains were grown in Luria–Bertani (LB) medium supplemented with the appropriate antibiotics, incubated at 37 °C, and shaken for liquid cultures.

### DNA Tools and Vectors

*S. hofmannii* Tn7-like transposase components including shCas12k, the operon encoding TnsB, TnsC, and TniQ, and the optimized sgRNA scaffold were obtained from pHelper_ShCAST; the left- and right-end (LE and RE) sequences of the Tn7-like transposon were obtained from pDonor_ShCAST.^29^ The pJ23119 promoter and T7Te terminator were PCR-amplified from pHelper_ShCAST together with the optimized single guide (sg) RNA scaffold sequence that contained the lgu1 sites for insertion of the target-specific spacer. P_*gln A*_ was amplified from the pRL3845 plasmid.^21^ All other promoters and terminators were obtained from the CyanoGate system.^25^ The spacer part of the sgRNA was assembled by the hybridization of two complementary synthetic oligonucleotides (Integrate DNA Technologies; Supporting Figure S2 and Table S4). The coding sequences for the erythromycin and Sm/Sp resistance genes were obtained from the CyanoGate system.^25^ For cloning into level 0 vectors and when necessary, restriction sites for the type IIS restriction enzymes *BsaI* and *BpiI* were removed from the above-mentioned sequences during PCR amplification (domesticated).

Vectors to assemble the plasmids to test CAST in cyanobacteria were obtained from the MoClo Plant Tool kit following the pipeline described in ref (37) where level 0 plasmids contain individual components (promoters, coding regions, terminators, etc.) and expression cassettes are assembled in level 1 plasmids. The final level T plasmids, containing all of the expression cassettes, were assembled using the replicative and conjugative vector pCAT.000 or the replicative but not conjugative vector pCAT.334 from the CyanoGate system.^25^ In addition, cyanobacterial replication encoded by OriT in pCAT.000 was replaced with ColE1 from pEERM3^38^ to obtain a T-level backbone allowing conjugation but not replication (committing suicide) in the cyanobacterial host.

### Plasmid Construct and Bacterial Colony Screening

All plasmids generated in this work were named pAzUX.Y (plasmid Azolla Utrecht), where X indicates the level and Y is the specific ID (Supporting Table S1). They will be submitted to Addgene (reference number upon acceptance of the manuscript) to ease sharing.

To obtain plasmids pAzU0.1, pAzU0.2, and pAzU0.3, sequences from the ShCAST components were PCR-amplified to remove internal *Bsa*I and *Bpi*I and introduce flanking *Bsa*I restriction sites. The coding sequences for the erythromycin and Sm/Sp resistance genes were also amplified to introduce the flanking *Bsa*I sites. In all cases, Phusion high-fidelity DNA polymerase (ThermoFisher) was used following the manufacturer’s instructions. All other individual components were obtained using CyanoGate or MoClo level 0 plasmids (Figure 1). Erythromycin selection was privileged in this study because the antibiotic does not affect the plant host such as, for example, in the symbioses of ferns from the genus *Azolla.*(39)

Level 1 plasmids were assembled by digestion with *Bsa*I and ligation, following the MoClo cloning protocol.^40^ To obtain pAzU1.3.3, the annealed oligonucleotide spacers were introduced in plasmid pAzU1.3 containing the sgRNA scaffold by *Lgu*I digestion and ligation (Figure 1). To obtain level T plasmids, level 1 inserts were introduced in pCAT.000 or pCAT.334, together with an end-linker (L), by *Bpi*I digestion and ligation. All restriction enzymes and T4-DNA ligase were from ThermoFisher.

Plasmids were introduced in *E. coli* DH5α and HB101 by heat shock. Putative positive colonies were selected using the appropriate antibiotics. Positive colonies were confirmed by PCR with Dream Taq polymerase using specific primers. Amplified fragments were purified and sequenced (Macrogen Europe).

### Cyanobacterial Transformation and Sonication of Exconjugants

*E. coli* strains ED8634 (containing pRL443 encoding the conjugation machinery) and HB101 (containing pRL623 and the cargo plasmid) were used for triparental conjugation as described in ref (36). The mixture of *E. coli* and cyanobacteria was spread and cultured on filters deposited for 24 h on plates with a solidified mixture of BG11 (95%) and LB (5%), and then transferred to BG11 medium for after another 24 h. Filters were moved to BG11 (or BG11_0_ for the rapid conjugation protocol) plates supplemented with Sm, Sp, and Em for 48 h, then transferred back to BG11 medium supplemented with the corresponding antibiotics. Potential positive colonies growing after 2 weeks were restreaked and confirmed by PCR amplification followed by sequencing, as described above.

To generate clonal strains of exconjugants, exconjugants that had integrated the YFP-encoding sequence into the genomic DNA were grown in 25 mL of BG11 medium to an optical density (OD) of 1 at 750 nm. Then, under sterile conditions, 1 mL of culture was removed and placed in a sterile plastic tube for sonication. During the sonication step, the filament length was monitored until most of the filaments were broken down to 2–3 cells. Finally, serial dilutions were made and 50 μL of each dilution was plated on solid BG11 medium supplemented with antibiotic resistance encoded on the transposon but without Km (the antibiotic resistance encoded on the plasmid backbone) and allowed to grow under the conditions described above. The genotypes of the clonal colonies thus obtained were analyzed by PCR (Supporting Table S4).

To test relative growth rates, cultures grown in liquid BG11 medium with the corresponding antibiotic for 1 week were washed with BG11_0_ medium so as to not carry over nitrogen when next plating on BG11_0_ medium, inoculated with the chlorophyll equivalents indicated and incubated in the light at 30 °C for 8, 12, and 28 days.

### Confocal Microscopy

Samples were grown on a plate of BG11 medium supplemented with the corresponding antibiotic under conditions previously described. Biomass was taken with a toothpick and suspended in 100 μL of sterile distilled water. For fluorescence detection, drops of 10 μL were placed on a new BG11 plate, cut out of the agar, and covered with a coverslip. Images were photographed using a Leica SP5 microscope (40× oil immersion objective). YFP was excited using a 514 nm laser and sf-GFP using a 488 nm laser; both lasers were used at 20% power and the irradiation came from an argon ion laser. The fluorescence of YFP was visualized with a window of 515–545 nm, and for sf-GFP, a window of 500–525 nm was used. Autofluorescence from the natural pigments of cyanobacterial cells was collected using a window of 640–740 nm. ImageJ software was then used to remove the background as well as the overlapping images.^41^

### DNA Extraction and Sequencing

Cyanobacterial genomic DNA was isolated using the GeneJET genomic DNA purification kit (ThermoFisher) following the manufacturer’s instructions. DNA quality and concentration were analyzed by UV absorption, q-bit, and on gel.

Minion sequencing libraries were generated using 0.3–3 μg of DNA with the SQK-LSK109 kit following instructions by Nanopore Technologies (version NBE_9065_v109_revAK_14Aug2019); in the case of multiplexing, the EXP-NBD104 extension was combined with NEB Blunt/TA ligase master mix (M0367, New England BioLabs (NEB)). Briefly, the DNA was first repaired (NEBNextFFPE repair mix (M6630) and NEBNext Ultra II End repair/dA-tailing module (E7546)), then bound on AMPure XP beads (Beckman & Coulter), and cleaned twice with 75% v/v ethanol before elution in water at 50 °C for 10 min. Subsequently, when barcoded, the barcode adapters were ligated and the DNA was cleaned once more using the AMPure XP beads and 75% v/v ethanol. Finally, the DNA was ligated to the sequencing primers in the presence of the tether and washed with short fragment buffer (SQK-LSK109 kit, Nanopore Technologies) bound once more on the AMPure XP beads before elution in the elution buffer at 50 °C for 10 min. Priming and loading of the recycled MinION flowcell (R9.4.1 Nanopore Technologies, starting with 600 active pores) were as per the manufactuer’s instruction using reagents from EXP-FLP002 (Nanopore Technologies). The flowcell was washed between the loading of different libraries using the EXP-WSH004 (Nanopore Technologies) reagents that included DNase.

### Genome Assemblies and Annotation

Data acquisition was done using the Minknow program (Oxford Nanopore Technologies) until 50 times genome coverage was achieved for the barcode with the lowest reads. The actual coverage ranged from 51 (UU1_1) to 233 (UU1_4), with two outliers 21 (CSAM) and 35 (UU2_3). Base calling was carried out separately. Assemblies were computed *de novo* using Flye^42^ with default settings, then visualized using Bandage;^43^ polishing using Medaka proved not to improve the assemblies; annotation of the assemblies was carried out with Bakta^44^ and Prokka^45^ for comparison. *Anabaena* genomes and alignment files of the minion reads aligned to them with Minimap2^46,^ were visualized using Integral Genome Viewer (IGV^47^). The BLAT function inside IGV was used to locate YFP, GFP, Amt1, and SepJ, as well as the left- and right-end sequences of Tn7 provided in the original plasmid pAzUT14. Large structural variations between the transconjugant after RNA-guided transposition and the reference genomes were programmatically detected using Sniffles 2, after mapping of the reads using NGMLR.^48^ The log of the analyses is detailed at https://github.com/lauralwd/anabaena_nanopore_workflow/blob/main/script.sh. Sniffles 2 is highly dependent on assembly quality and we therefore show results for assemblies with the highest coverage. When comparing all of the strains with the transposon targeted into the *amt1::gfp* locus, Sniffles 2 identified 15 insertions, three deletions, and four recombinations (Supporting Table S3); 13 putative events were in the plasmids. Given the higher fluidity with which plasmids were assembled, we suspected oversampling during assembly, but the read coverage for the plasmids was similar to that of the chromosome. The indels were then inspected by extracting the FASTA files defined by their boundaries in Sniffles 2, then locating their regions in the assemblies of the transconjugants using BLAT in IGV for further evaluation (Supporting Figures 5–12).

## Data Availability

CASTGATE vectors listed in Supporting Table S1 have been deposited with Addgene (reference numbers provided upon manuscript acceptance). Nanopore (MinION) sequencing data were deposited at the European Nucleotide Archive (ENA) and are made available under the accession number PRJEB60371.

## Supporting Information Available

Relative toxicity of the CAST plasmids in *Anabaena* (Figure S1); the three different sgRNAs targeting the GFP (Figure S2); efficient targeting at locus alr3727 in wild-type *Anabaena* (Figure S3); rapid conjugation protocol for RNA-guided transposition using the suicide plasmid pAzUT.17 (Figure S4); figures S5–S12; visualization of the loci of candidate Indels listed in Supporting Table S3; indels were identified programmatically (with Sniffles_2) comparing the parental CSVT15 with their respective transconjugant strains obtained after RNA-guided transposition events UU1-1, UU1-2, and UU1-3; CASTGATE vectors generated in this study (Table S1); CASTGATE vectors transferred to and tested in wild-type *Anabaena*, and the CSVT15 and CSAM137 strains in this study (Table S2); insertions detected by Sniffles comparing genome assemblies from the parental strains with those from clones obtained after RNA-guided transposition (Table S3); primers used for PCR assays and key cloning steps in this study (Table S4) (PDF)

## References

[ref1] Sánchez-Baracaldo P.; Bianchini G.; Wilson J. D.; Knoll A. H. Cyanobacteria and Biogeochemical Cycles through Earth History. Trends Microbiol. 2022, 30 (2), 143–157. 10.1016/j.tim.2021.05.008. 34229911

[ref2] Zehr J. P.; Capone D. G. Changing Perspectives in Marine Nitrogen Fixation. Science 2020, 368 (6492), eaay9514 10.1126/science.aay9514. 32409447

[ref3] Mutalipassi M.; Riccio G.; Mazzella V.; Galasso C.; Somma E.; Chiarore A.; De Pascale D.; Zupo V. Symbioses of Cyanobacteria in Marine Environments: Ecological Insights and Biotechnological Perspectives. Mar. Drugs 2021, 19 (4), 227 10.3390/md19040227. 33923826 PMC8074062

[ref4] Rikkinen J. Cyanobacteria in Terrestrial Symbiotic Systems. In Modern Topics in the Phototrophic Prokaryotes: Environmental and Applied Aspects; Springer, 2017; pp 243–294 10.1007/978-3-319-46261-5_8.

[ref5] Gutiérrez S.; Lauersen K. J. Gene Delivery Technologies with Applications in Microalgal Genetic Engineering. Biology 2021, 10 (4), 265 10.3390/BIOLOGY10040265. 33810286 PMC8067306

[ref6] Elhai J.; Vepritskiy A.; Muro-Pastor A. M.; Flores E.; Wolk C. P. Reduction of Conjugal Transfer Efficiency by Three Restriction Activities of Anabaena Sp. Strain PCC 7120. J. Bacteriol. 1997, 179 (6), 1998–2005. 10.1128/jb.179.6.1998-2005.1997. 9068647 PMC178925

[ref7] Baldanta S.; Guevara G.; Navarro-Llorens J. M. SEVA-Cpf1, a CRISPR-Cas12a Vector for Genome Editing in Cyanobacteria. Microb. Cell Fact. 2022, 21 (1), 103 10.1186/s12934-022-01830-4. 35643551 PMC9148489

[ref8] Ungerer J.; Pakrasi H. B. Cpf1 Is A Versatile Tool for CRISPR Genome Editing Across Diverse Species of Cyanobacteria. Sci. Rep. 2016, 6, 39681 10.1038/srep39681. 28000776 PMC5175191

[ref9] Niu T. C.; Lin G. M.; Xie L. R.; Wang Z. Q.; Xing W. Y.; Zhang J. Y.; Zhang C. C. Expanding the Potential of CRISPR-Cpf1-Based Genome Editing Technology in the Cyanobacterium Anabaena PCC 7120. ACS Synth. Biol. 2019, 8 (1), 170–180. 10.1021/acssynbio.8b00437. 30525474

[ref10] Klompe S. E.; Vo P. L. H.; Halpin-Healy T. S.; Sternberg S. H. Transposon-Encoded CRISPR–Cas Systems Direct RNA-Guided DNA Integration. Nature 2019, 571 (7764), 219–225. 10.1038/s41586-019-1323-z. 31189177

[ref11] Peters J. E.; Craig N. L. Tn7 Recognizes Transposition Target Structures Associated with DNA Replication Using the DNA-Binding Protein TnsE. Genes Dev. 2001, 15 (6), 737–747. 10.1101/gad.870201. 11274058 PMC312648

[ref12] González Linares R. A CRISPR-Associated Transposase Presents Null Cargo Integration Efficiency When Targeting a Transcriptionally Highly Active Region. 2020.

[ref13] Bialk P.; Rivera-Torres N.; Strouse B.; Kmiec E. B. Regulation of Gene Editing Activity Directed by Single-Stranded Oligonucleotides and CRISPR/Cas9 Systems. PLoS One 2015, 10 (6), e0129308 10.1371/journal.pone.0129308. 26053390 PMC4459703

[ref14] Zhang S.; Guo F.; Yan W.; Dai Z.; Dong W.; Zhou J.; Zhang W.; Xin F.; Jiang M. Recent Advances of CRISPR/Cas9-Based Genetic Engineering and Transcriptional Regulation in Industrial Biology. Front. Bioeng. Biotechnol. 2020, 7, 459 10.3389/fbioe.2019.00459. 32047743 PMC6997136

[ref15] Querques I.; Schmitz M.; Oberli S.; Chanez C.; Jinek M. Target Site Selection and Remodelling by Type V CRISPR-Transposon Systems. Nature 2021, 599 (7885), 497–502. 10.1038/s41586-021-04030-z. 34759315 PMC7613401

[ref16] Stellwagen A. E.; Craig N. L. Avoiding Self: Two Tn7-Encoded Proteins Mediate Target Immunity in Tn7 Transposition. EMBO J. 1997, 16 (22), 6823–6834. 10.1093/emboj/16.22.6823. 9362496 PMC1170286

[ref17] Hoffmann F. T.; Kim M.; Beh L. Y.; Wang J.; Vo P. L. H.; Gelsinger D. R.; George J. T.; Acree C.; Mohabir J. T.; Fernández I. S.; Sternberg S. H. Selective TnsC Recruitment Enhances the Fidelity of RNA-Guided Transposition. Nature 2022, 609 (7926), 384–393. 10.1038/s41586-022-05059-4. 36002573 PMC10583602

[ref18] Peters J. E.; Craig N. L. Tn7: Smarter than We Thought. Nat. Rev. Mol. Cell Biol. 2001, 2 (11), 806–814. 10.1038/35099006. 11715047

[ref19] Park J. U.; Tsai A. W. L.; Mehrotra E.; Petassi M. T.; Hsieh S. C.; Ke A.; Peters J. E.; Kellogg E. H. Structural Basis for Target Site Selection in RNA-Guided DNA Transposition Systems. Science 2021, 373 (6556), 768–774. 10.1126/science.abi8976. 34385391 PMC9080059

[ref20] Faure G.; Shmakov S. A.; Yan W. X.; Cheng D. R.; Scott D. A.; Peters J. E.; Makarova K. S.; Koonin Ev. CRISPR–Cas in Mobile Genetic Elements: Counter-Defence and Beyond. Nat. Rev. Microbiol. 2019, 17 (8), 513–525. 10.1038/s41579-019-0204-7. 31165781 PMC11165670

[ref21] Valladares A.; Muro-Pastor A. M.; Herrero A.; Flores E. The NtcA-Dependent P1 Promoter Is Utilized for GlnA Expression in N2-Fixing Heterocysts of Anabaena Sp. Strain PCC 7120. J. Bacteriol. 2004, 186 (21), 7337–7343. 10.1128/JB.186.21.7337-7343.2004. 15489445 PMC523192

[ref22] Merino-Puerto V.; Mariscal V.; Mullineaux C. W.; Herrero A.; Flores E. Fra Proteins Influencing Filament Integrity, Diazotrophy and Localization of Septal Protein SepJ in the Heterocyst-Forming Cyanobacterium Anabaena Sp. Mol. Microbiol. 2010, 75 (5), 1159–1170. 10.1111/j.1365-2958.2009.07031.x. 20487302

[ref23] Flores E.; Pernil R.; Muro-Pastor A. M.; Mariscal V.; Maldener I.; Lechno-Yossef S.; Fan Q.; Wolk C. P.; Herrero A. Septum-Localized Protein Required for Filament Integrity and Diazotrophy in the Heterocyst-Forming Cyanobacterium Anabaena Sp. Strain PCC 7120. J. Bacteriol. 2007, 189 (10), 3884–3890. 10.1128/JB.00085-07. 17369306 PMC1913322

[ref24] Hu B.; Yang G.; Zhao W.; Zhang Y.; Zhao J. MreB Is Important for Cell Shape but Not for Chromosome Segregation of the Filamentous Cyanobacterium Anabaena Sp. PCC 7120. Mol. Microbiol. 2007, 63 (6), 1640–1652. 10.1111/j.1365-2958.2007.05618.x. 17367385

[ref25] Vasudevan R.; Gale G. A. R.; Schiavon A. A.; Puzorjov A.; Malin J.; Gillespie M. D.; Vavitsas K.; Zulkower V.; Wang B.; Howe C. J.; Lea-Smith D. J.; McCormick A. J. CyanoGate: A Modular Cloning Suite for Engineering Cyanobacteria Based on the Plant MoClo Syntax. Plant Physiol. 2019, 180 (1), 39–55. 10.1104/pp.18.01401. 30819783 PMC6501082

[ref26] Rubin B. E.; Diamond S.; Cress B. F.; Crits-Christoph A.; Lou Y. C.; Borges A. L.; Shivram H.; He C.; Xu M.; Zhou Z.; Smith S. J.; Rovinsky R.; Smock D. C. J.; Tang K.; Owens T. K.; Krishnappa N.; Sachdeva R.; Barrangou R.; Deutschbauer A. M.; Banfield J. F.; Doudna J. A. Species- and Site-Specific Genome Editing in Complex Bacterial Communities. Nat. Microbiol. 2022, 7 (1), 34–47. 10.1038/s41564-021-01014-7. 34873292 PMC9261505

[ref27] Xiao R.; Wang S.; Han R.; Li Z.; Gabel C.; Mukherjee I. A.; Chang L. Structural Basis of Target DNA Recognition by CRISPR-Cas12k for RNA-Guided DNA Transposition. Mol. Cell 2021, 81 (21), 4457–4466. 10.1016/J.MOLCEL.2021.07.043. 34450043 PMC8571069

[ref28] Heler R.; Samai P.; Modell J. W.; Weiner C.; Goldberg G. W.; Bikard D.; Marraffini L. A. Cas9 Specifies Functional Viral Targets during CRISPR–Cas Adaptation. Nature 2015, 519 (7542), 199–202. 10.1038/nature14245. 25707807 PMC4385744

[ref29] Strecker J.; Ladha A.; Gardner Z.; Schmid-Burgk J. L.; Makarova K. S.; Koonin E. V.; Zhang F. RNA-Guided DNA Insertion with CRISPR-Associated Transposases. Science 2019, 365 (6448), 48–53. 10.1126/science.aax9181. 31171706 PMC6659118

[ref30] Saito M.; Ladha A.; Strecker J.; Faure G.; Neumann E.; Altae-Tran H.; Macrae R. K.; Zhang F. Dual Modes of CRISPR-Associated Transposon Homing. Cell 2021, 184 (9), 2441–2453.e18. 10.1016/J.CELL.2021.03.006. 33770501 PMC8276595

[ref31] Kaczmarska Z.; Czarnocki-Cieciura M.; Górecka-Minakowska K. M.; Wingo R. J.; Jackiewicz J.; Zajko W.; Poznański J. T.; Rawski M.; Grant T.; Peters J. E.; Nowotny M. Structural Basis of Transposon End Recognition Explains Central Features of Tn7 Transposition Systems. Mol. Cell 2022, 82 (14), 2618–2632.e7. 10.1016/j.molcel.2022.05.005. 35654042 PMC9308760

[ref32] Vo P. L. H.; Acree C.; Smith M. L.; Sternberg S. H. Unbiased Profiling of CRISPR RNA-Guided Transposition Products by Long-Read Sequencing. Mobile DNA 2021, 12 (1), 13 10.1186/s13100-021-00242-2. 34103093 PMC8188705

[ref33] Vo P. L. H.; Ronda C.; Klompe S. E.; Chen E. E.; Acree C.; Wang H. H.; Sternberg S. H. CRISPR RNA-Guided Integrases for High-Efficiency, Multiplexed Bacterial Genome Engineering. Nat. Biotechnol. 2021, 39 (4), 480–489. 10.1038/s41587-020-00745-y. 33230293 PMC10583764

[ref34] Tou C. J.; Orr B.; Kleinstiver B. P. Cut-and-Paste DNA Insertion with Engineered Type V-K CRISPR-Associated Transposases bioRxiv 2022, p 2022-01 10.1101/2022.01.07.475005. 36593413

[ref35] Rippka R.; Waterbury J.; Cohen-Bazire G. A Cyanobacterium Which Lacks Thylakoids. Arch. Microbiol. 1974, 100 (1), 419–436. 10.1007/BF00446333.

[ref36] Elhai J.; Wolk C. P. [83] Conjugal Transfer of DNA to Cyanobacteria. Methods Enzymol. 1988, 167 (C), 747–754. 10.1016/0076-6879(88)67086-8. 3148842

[ref37] Engler C.; Youles M.; Gruetzner R.; Ehnert T. M.; Werner S.; Jones J. D. G.; Patron N. J.; Marillonnet S. A Golden Gate Modular Cloning Toolbox for Plants. ACS Synth. Biol. 2014, 3 (11), 839–843. 10.1021/sb4001504. 24933124

[ref38] Englund E.; Andersen-Ranberg J.; Miao R.; Hamberger B.; Lindberg P. Metabolic Engineering of Synechocystis Sp. PCC 6803 for Production of the Plant Diterpenoid Manoyl Oxide. ACS Synth. Biol. 2015, 4 (12), 1270–1278. 10.1021/acssynbio.5b00070. 26133196 PMC4685428

[ref39] Dijkhuizen L. W.; Brouwer P.; Bolhuis H.; Reichart G. J.; Koppers N.; Huettel B.; Bolger A. M.; Li F. W.; Cheng S.; Liu X.; Wong G. K. S.; Pryer K.; Weber A.; Bräutigam A.; Schluepmann H. Is There Foul Play in the Leaf Pocket? The Metagenome of Floating Fern Azolla Reveals Endophytes That Do Not Fix N2 but May Denitrify. New Phytol. 2018, 217 (1), 453–466. 10.1111/nph.14843. 29084347 PMC5901025

[ref40] Weber E.; Engler C.; Gruetzner R.; Werner S.; Marillonnet S. A Modular Cloning System for Standardized Assembly of Multigene Constructs. PLoS One 2011, 6 (2), e16765 10.1371/journal.pone.0016765. 21364738 PMC3041749

[ref41] Schindelin J.; Rueden C. T.; Hiner M. C.; Eliceiri K. W. The ImageJ Ecosystem: An Open Platform for Biomedical Image Analysis. Mol. Reprod. Dev. 2015, 82 (7–8), 518–529. 10.1002/mrd.22489. 26153368 PMC5428984

[ref42] Kolmogorov M.; Yuan J.; Lin Y.; Pevzner P. A. Assembly of Long, Error-Prone Reads Using Repeat Graphs. Nat. Biotechnol. 2019, 37 (5), 540–546. 10.1038/s41587-019-0072-8. 30936562

[ref43] Wick R. R.; Schultz M. B.; Zobel J.; Holt K. E. Bandage: interactive visualization of de novo genome assemblies. Bioinformatics 2015, 31 (20), 3350–3352. 10.1093/bioinformatics/btv383. 26099265 PMC4595904

[ref44] Schwengers O.; Jelonek L.; Dieckmann M. A.; Beyvers S.; Blom J.; Goesmann A. Bakta: Rapid and Standardized Annotation of Bacterial Genomes via Alignment-Free Sequence Identification. Microb. Genomics 2021, 7 (11), 000685 10.1099/mgen.0.000685. PMC8743544 34739369

[ref45] Seemann T. Prokka: Rapid Prokaryotic Genome Annotation. Bioinformatics 2014, 30 (14), 2068–2069. 10.1093/bioinformatics/btu153. 24642063

[ref46] Li H. Minimap2: Pairwise Alignment for Nucleotide Sequences. Bioinformatics 2018, 34 (18), 3094–3100. 10.1093/bioinformatics/bty191. 29750242 PMC6137996

[ref47] Thorvaldsdóttir H.; Robinson J. T.; Mesirov J. P. Integrative Genomics Viewer (IGV): High-Performance Genomics Data Visualization and Exploration. Briefings Bioinf. 2013, 14 (2), 178–192. 10.1093/bib/bbs017. PMC3603213 22517427

[ref48] Sedlazeck F. J.; Rescheneder P.; Smolka M.; Fang H.; Nattestad M.; von Haeseler A.; Schatz M. C. Accurate Detection of Complex Structural Variations Using Single-Molecule Sequencing. Nat. Methods 2018, 15 (6), 461–468. 10.1038/s41592-018-0001-7. 29713083 PMC5990442

