# Supplementary material for: Genome Engineering by RNA-Guided Transposition for Anabaena sp. PCC 7120
Source: ACS Synth Biol. 2024 Mar 6;13(3):901–12. doi: 10.1021/acssynbio.3c00583 (PMC10949235; doi:10.1021/acssynbio.3c00583)
Supplement: Supplementary file 1 — sb3c00583_si_001.pdf [file sb3c00583_si_001.pdf]

Supplemental data for the article:

Title: Genome engineering by RNA-guided transposition for *Anabaena* PCC 7120

Authors:

Sergio Arévalo[1,2,3,4], Daniel Pérez Rico[1], María Dolores Abarca[1,5], Laura Dijkhuizen[1], Cristina Sarasa-Buisan[3], Peter Lindblad[2], Enrique Flores[3], Sandra Nierzwicki-Bauer[4] and Henriette Schluepmann[1]

Affiliations:

[1] Biology Department, Utrecht University, Utrecht, The Netherlands.

[2] Microbial chemistry, Department of Chemistry-Ångström Laboratory, Uppsala University, Uppsala, Sweden.

[3] Instituto de Bioquímica Vegetal y Fotosíntesis, CSIC and Universidad de Sevilla, Seville, Spain.

[4] Department of Biological Sciences, Rensselaer Polytechnic Institute, Troy, NY, USA.

Current affiliation DA:

[5] Department of Life Sciences, University of Alcalá, Alcalá de Henares, Spain.

The following supplemental data is available in the online version of this article.

Supplemental Figure S1. Relative toxicity of the CAST plasmids in *Anabaena*.

Supplemental Figure S2. The three different sgRNAs targeting *gfp*.

Supplemental Figure S3. Efficient targeting at locus *alr3727* in wild-type *Anabaena*.

Supplemental Figure S4. Rapid conjugation protocol for RNA-guided transposition using the suicide plasmid pAzUT.17.

Supplemental Figures S5-S12. Visualization of the loci of candidate Indels listed in Supplemental Table S3. Indels were identified programmatically (with Sniffles_2) comparing the parental CSVT15 with their respective transconjugant strains obtained after RNA-guided transposition events UU1-1, UU1-2, UU1-3.

Supplemental Table S1. CASTGATE vectors generated in this study.

Supplemental Table S2. CASTGATE vectors transferred to and tested in *Anabaena* wild-type, and the CSVT15 and CSAM137 strains in this study.

Supplemental Table S3. Insertions detected by Sniffles comparing genome assemblies from the parental strains with those from clones obtained after RNA-guided transposition.

Supplemental Table S4. Primers used for PCR assays and key cloning steps in this study.

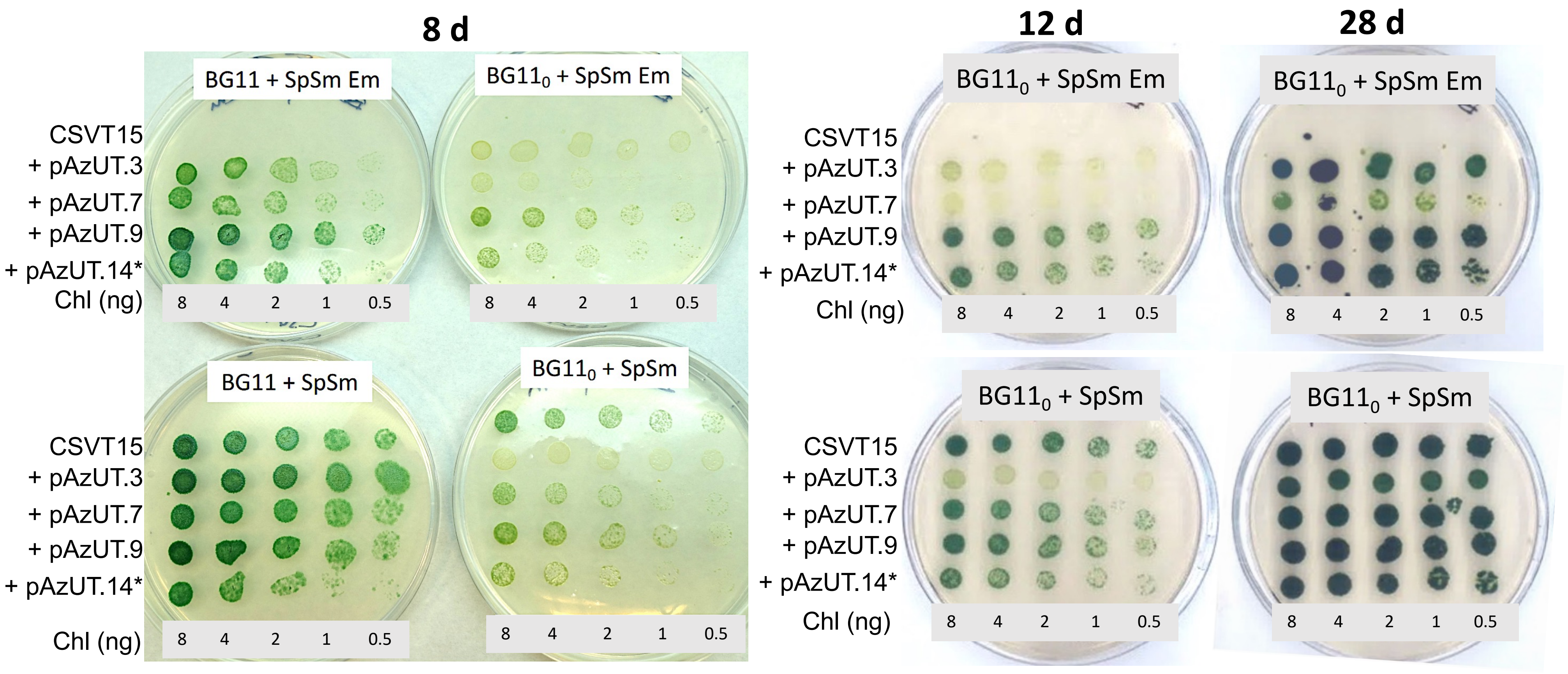

Supplemental Figure S1 Relative toxicity of the CAST plasmids in *Anabaena*. Exconjugant strains derived from CSVT15 generated by conjugation contained the following constructs: pAzUT.3, with only the YFP and erythromycin-resistance cassettes expressed constitutively; pAzUT.7 with, in addition to the components of pAzUT.3, the P_{glnA}-driven *cas12k* expression induced under nitrogen deprivation, and the strongly and constitutively expressed sgRNA scaffold (without the target specific sequence); pAzUT.9, with P_{glnA}-driven expression of all the CAST genes (*tnsB*, *tnsC*, *tniQ*, and *cas12k*), the cargo transposon flanked by the LE and RE, and the strongly and constitutively expressed sgRNA-scaffold without a target sequence. The strain carrying pAzUT.14*, used as a control, was homozygous for the cargo transposon inserted into the *gfp* and was cured of the pAzUT.14 plasmid that contained, in addition to what is encoded by pAzUT.9, the sgRNA targeting sense sequence of *gfp*. Strains with the plasmids and the controls, the parental strain and pAzUT.14 were grown in successive dilutions of starting material, measured in chlorophyll *a* equivalents (Chl in ng), for 8, 12 and 28 days on either Streptomycin/ Spectinomycin (Sm/Sp) or Sm/Sp and Erythromycin (Em) antibiotic selection in BG11 medium (with nitrogen) and BG11$_0$ medium (without combined nitrogen).

A

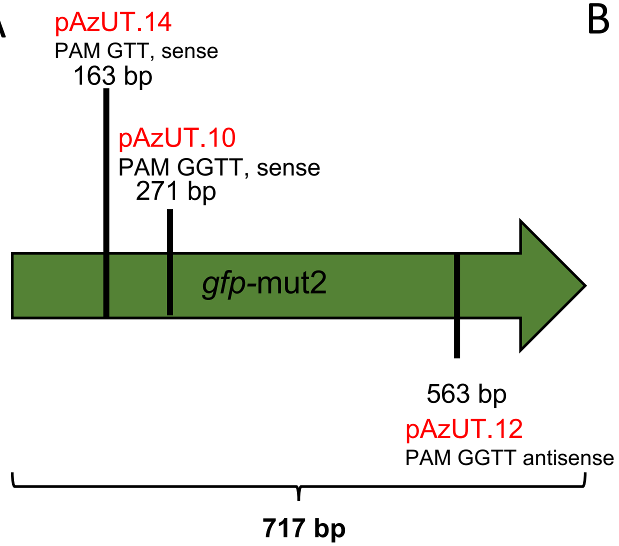

B

p label	Primer Orientation	Sequence	PAM	Target Strand
pAzUT.10	Fwd	TTGCTCTTCCAAAGGTTATGTACAGGAAAGAACTATATTTTTCAAAGGCTGGAAGAGCAA	GGTT	Sense
	Rev	TTGCTCTTCCAGCCTTTGAAAAATATAGTTCTTTCCTGTACATAACCTTTGGAAGAGCAA		
pAzUT.12	Fwd	TTGCTCTTCCAAAGGTTGTCTGGTAAAAGGACAGGGCCATCGCCAATGCTGGAAGAGCAA	GGTT	Antisense
	Rev	TTGCTCTTCCAGCATTGGCGATGGCCCTGTCCTTTTACCAGACAACCTTTGGAAGAGCAA		
pAzUT.14	Fwd	TTGCTCTTCCAAAGTTCCATGGCCAACACTTGTCACTACTTTCGCGGCTGGAAGAGCAA	GTT	Sense
	Rev	TTGCTCTTCCAGCCGCGAAAGTAGTGACAAGTGTTGGCCATGGAACTTTGGAAGAGCAA		

Supplemental Figure S2 The three different sgRNAs targeting *gfp*. A, Schematic representation of the coding region of *gfp-mut2* showing the base at which the different target-specific sequences start that are encoded by the sgRNA. For example, the target specific sequence of the sgRNA encoded in pAzUT.14 starts 163 bp inside the 717 bp-long *gfp-mut2* sequence with the GTT PAM sequence and encodes the sense strand. B, Primers used for cloning of the target specific sequences into the LguI restriction site of the scaffold sgRNA from pAzU1.3 (depicted in Fig.1). Forward (Fwd) and reverse (Rev) primers were annealed, then in one pot with pAzU1.3, digested and ligated to obtain the target specific sgRNA from the pAzUT.10, 12 and 14 as indicated. LguI restriction site (red), orientation specific overhangs (green), PAM (blue).

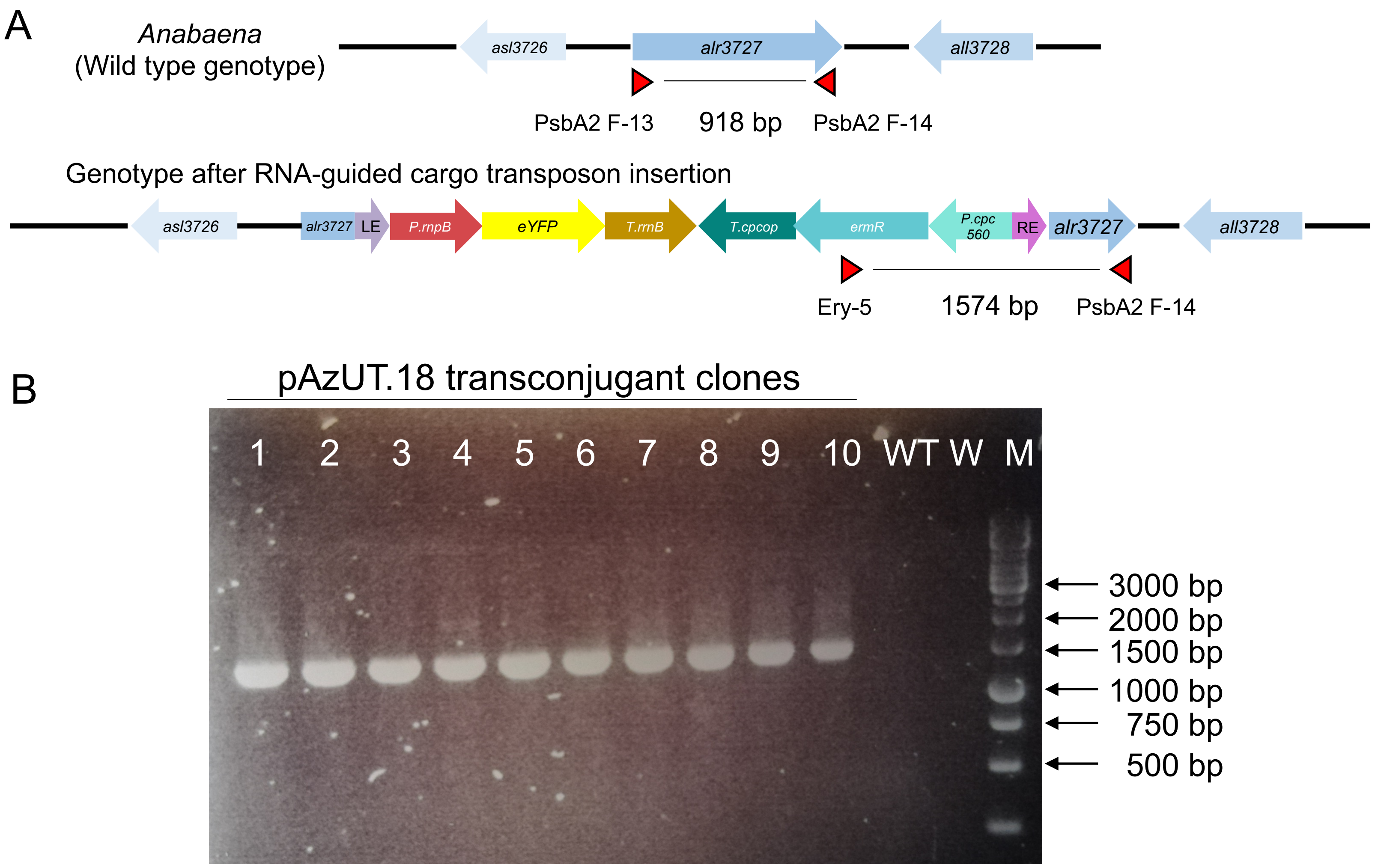

Supplemental Figure S3 Efficient targeting at locus *alr3727* in wild-type *Anabaena*. A, PCR strategy to detect RNA-guided transposition of the cargo transposon encoded in pAzUT.18 into the locus alr3727 of *Anabaena* sp. PCC 7120. (B) Fragments amplified from transconjugant clones obtained using a rapid process as follows: conjugation, subsequent transfers for 48 h on $BG11_0$ medium, followed by sonication and then selection on $BG11_0$ medium with erythromycin. 1-10, randomly selected transconjugant clones; WT, *Anabaena* sp. PCC 7120; W, water (negative control); M, DNA molecular weight standards.

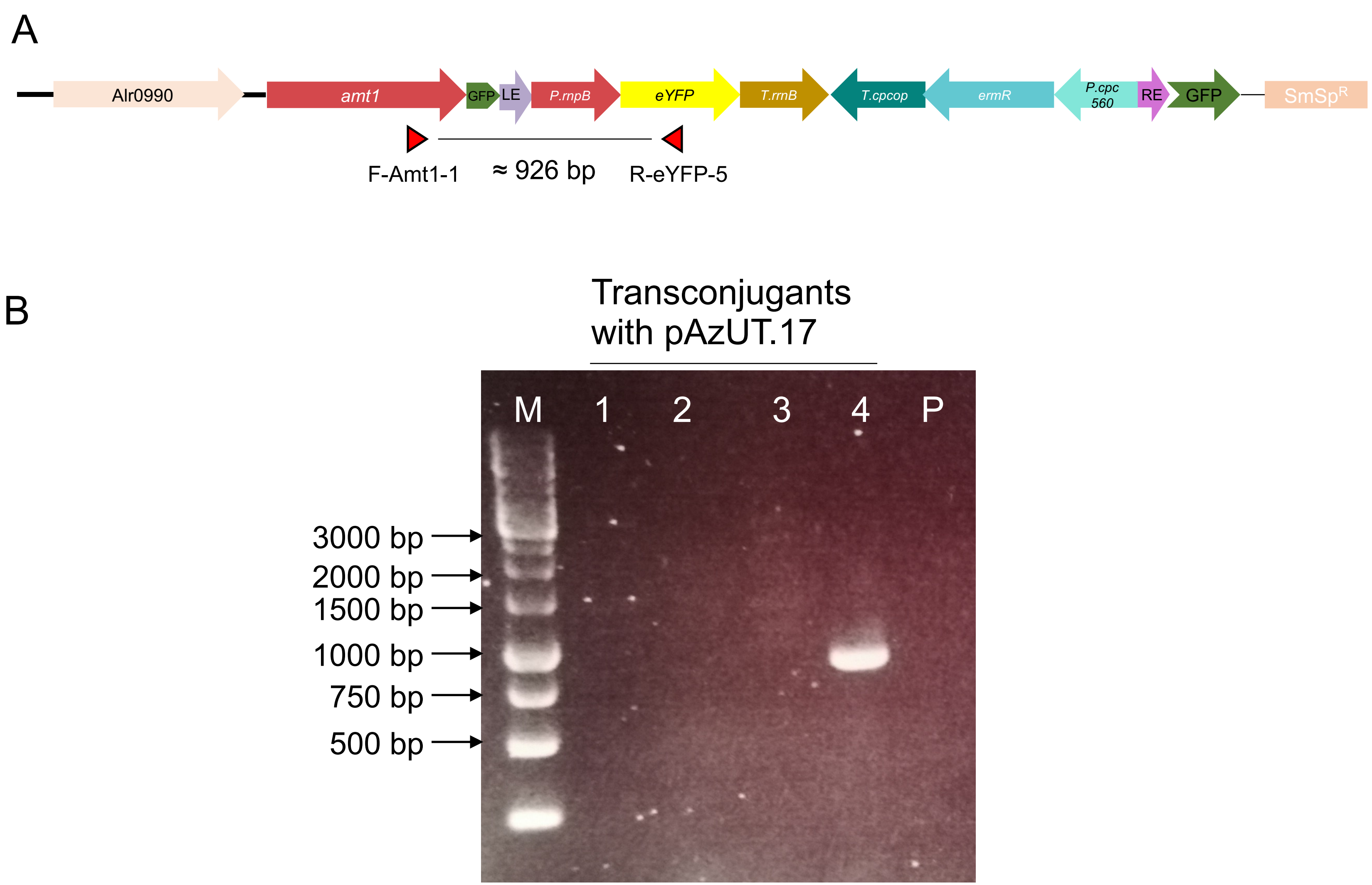

Supplemental Figure S4 Rapid conjugation protocol for RNA-guided transposition using the suicide plasmid pAzUT.17. pAzUT.17 is identical to pAzUT.14 except in the plasmid backbone region where the cyanobacterial replication elements were removed. In the rapid conjugation protocol with the parental strain CSV15, material spread in a filter on $BG11_0$ medium was transferred to $BG11_0$ medium during the first 48 h after of conjugation. Thereafter, transconjugants were selected on BG11 medium supplemented with erythromycin. A, Scheme of the *gfp* after insertion of RNA-guided cargo transposon and the PCR used to detect the insertion. B, PCR detection of the insertion at the LE of the cargo transposon. M, DNA molecular weight markers; 1-4, transconjugants with pAzUT.17 derived from CSV15 ; P, parental strain, CSVT15 (*amt1::gfp*).

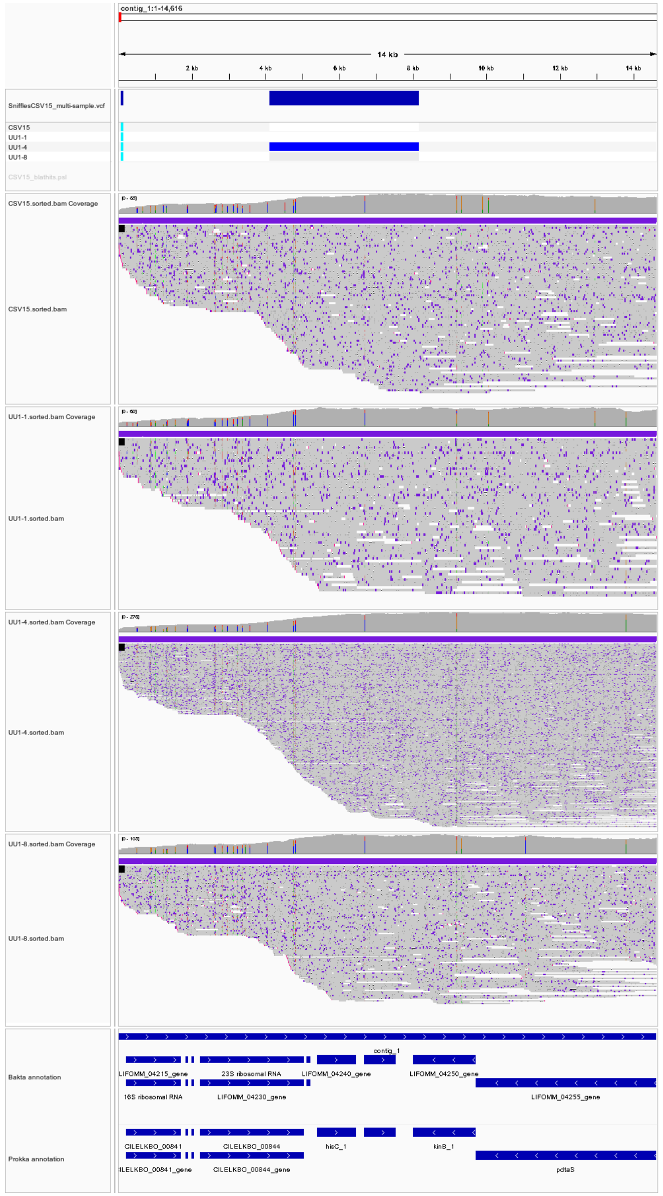

Supplemental Figure S5 Visualization in IGV of the chromosome loci for Indels 1 (contig_1_86_Sniffles2_INS_0M4) and 2 (contig_1_4114_Sniffles2_INS_1M4). These insertions are at the border of the chromosome and have no distinct border, therefore, they are a likely result of assembly issues at the end of the chromosome.

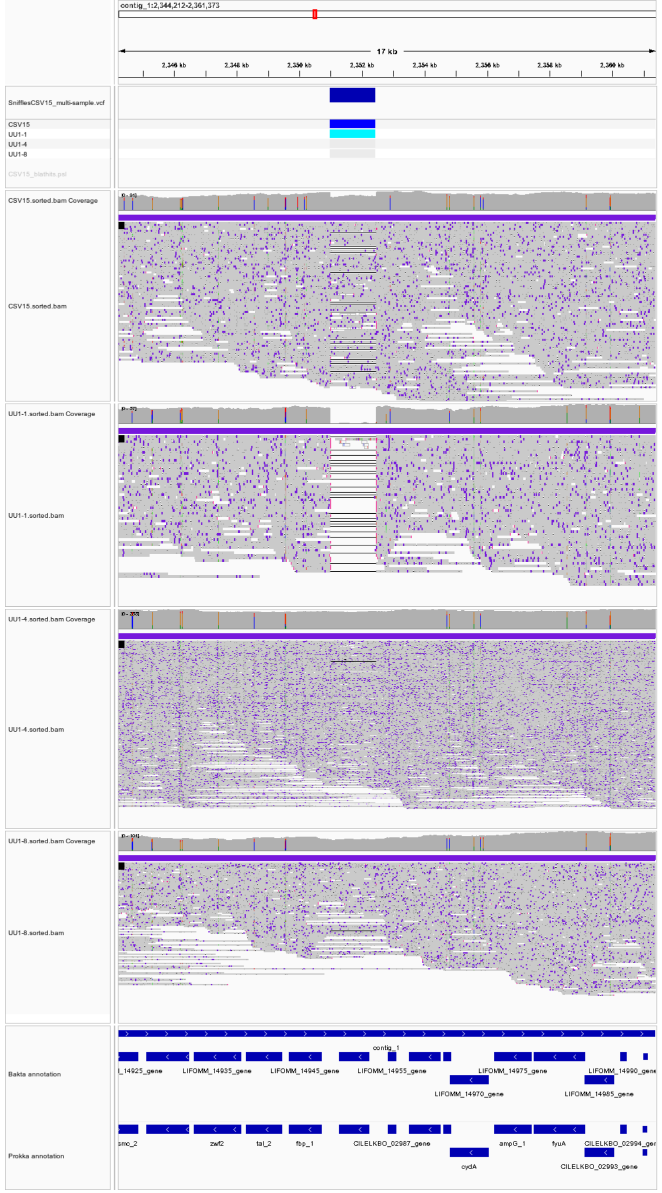

Supplemental Figure S6 Visualization in IGV of the locus for indel 3 (contig_1_2350996_Sniffels2.DEL_4M4). The deletion is already partially found in the parental strain CSVT15; it is dominant in the UU1_1 but barely detectable in the UU1_4 or UU1_8. This indel suggests relative instability of the chromosome of *Anabaena*, consistent with our finding when comparing the genome assemblies from CSVT15 and wt (data not shown).

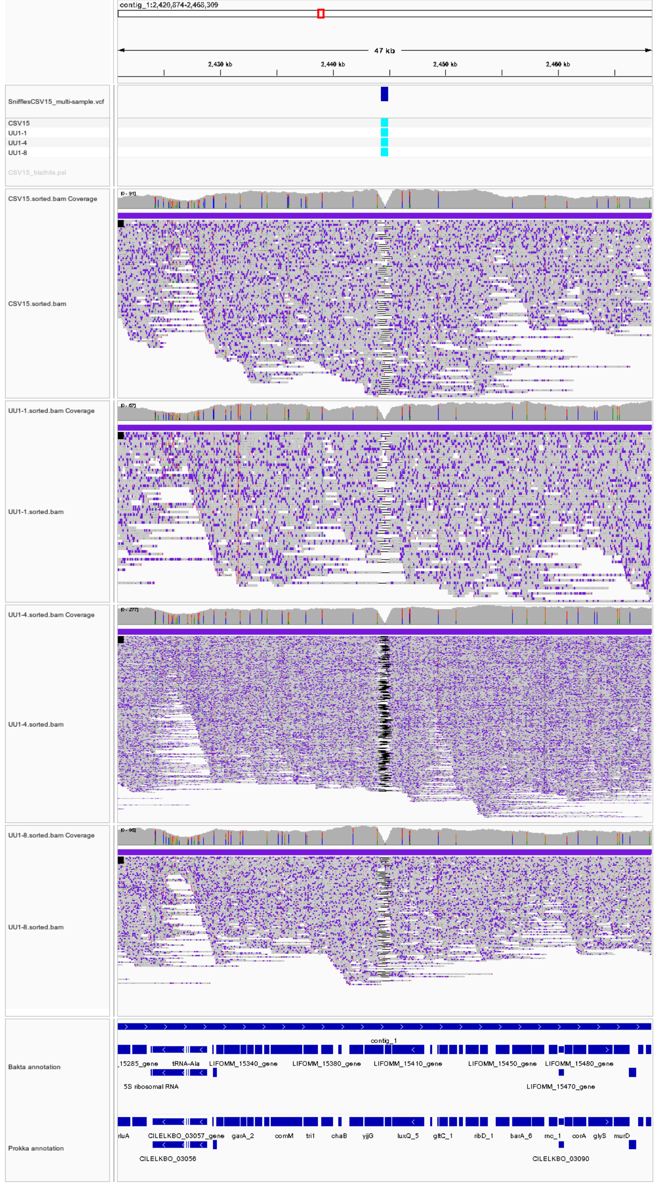

Supplemental Figure S7 Visualization in IGV of the locus for indel 4 (contig_1_2444322_Sniffles2_DEL_5M4). The v-shape lack of coverage for this deletion in all strains sequenced suggests a problem with assembly of the chromosome in the parental strain, possibly due to a region difficult to sequence. False positive indel.

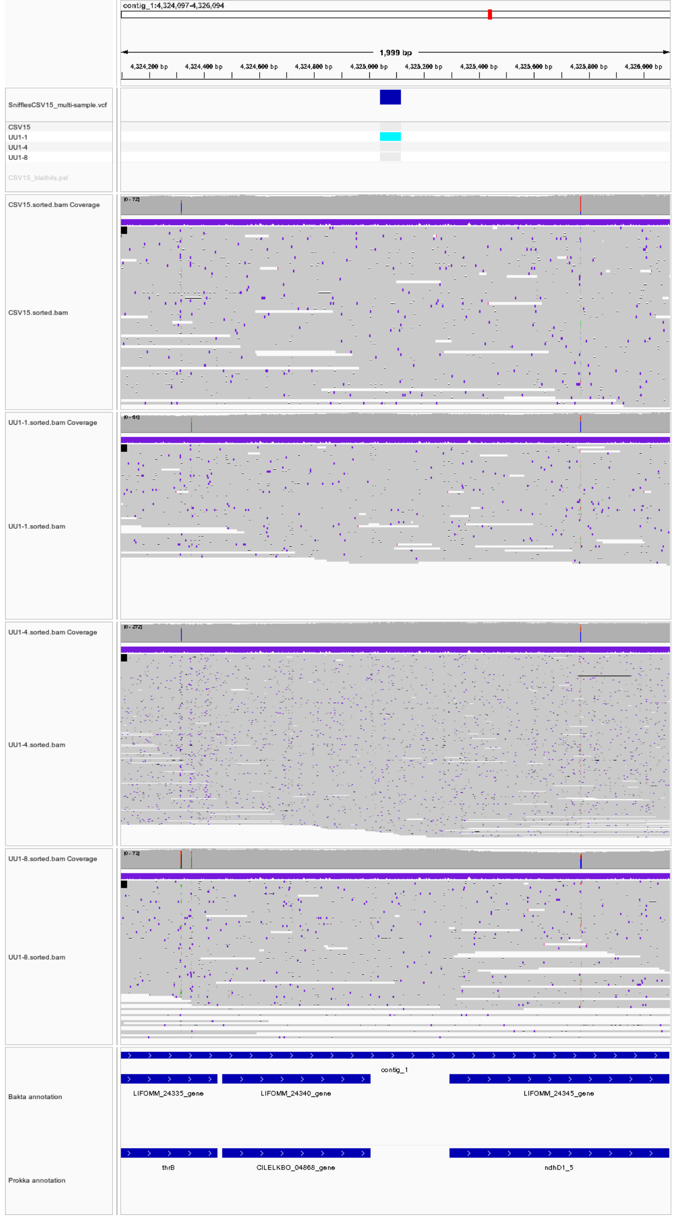

Supplemental Figure S8 Visualization in IGV of the locus for indel 5 (contig_1_4325042_Sniffles2_INS_6M4). Only a very minor proportion of the reads support the recombination in this region with differing end points, false positive indel thus.

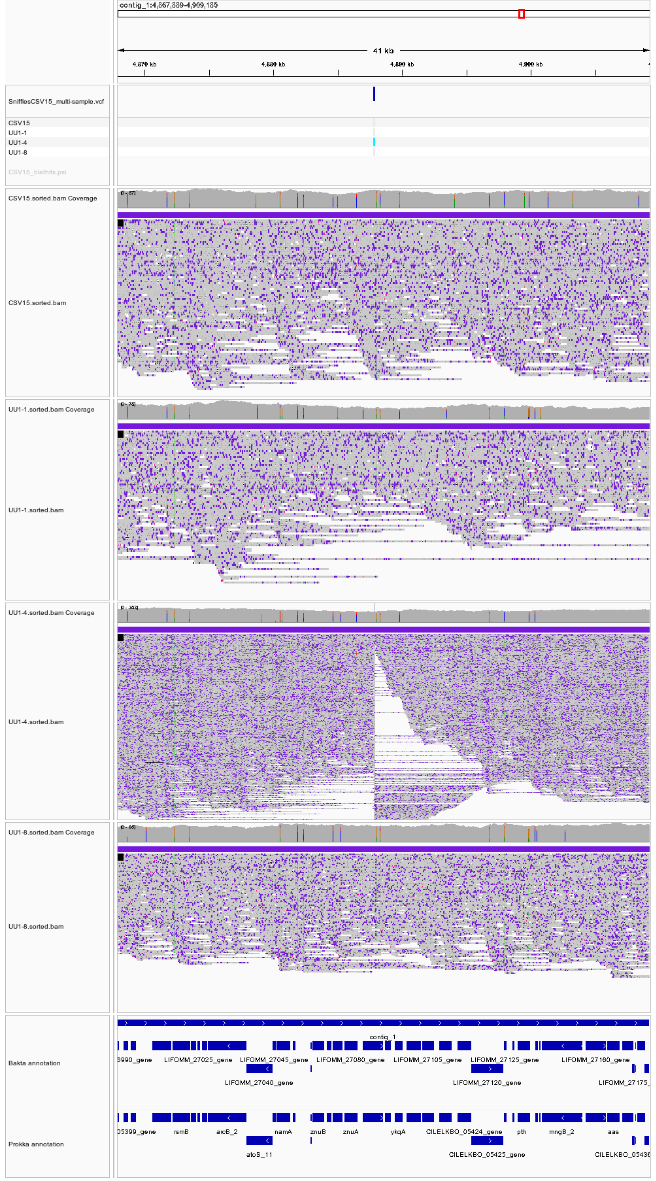

Supplemental Figure S9 Visualization in IGV of the locus for indel 6 (contig_1_4886060_Sniffles2_BND_8M4).

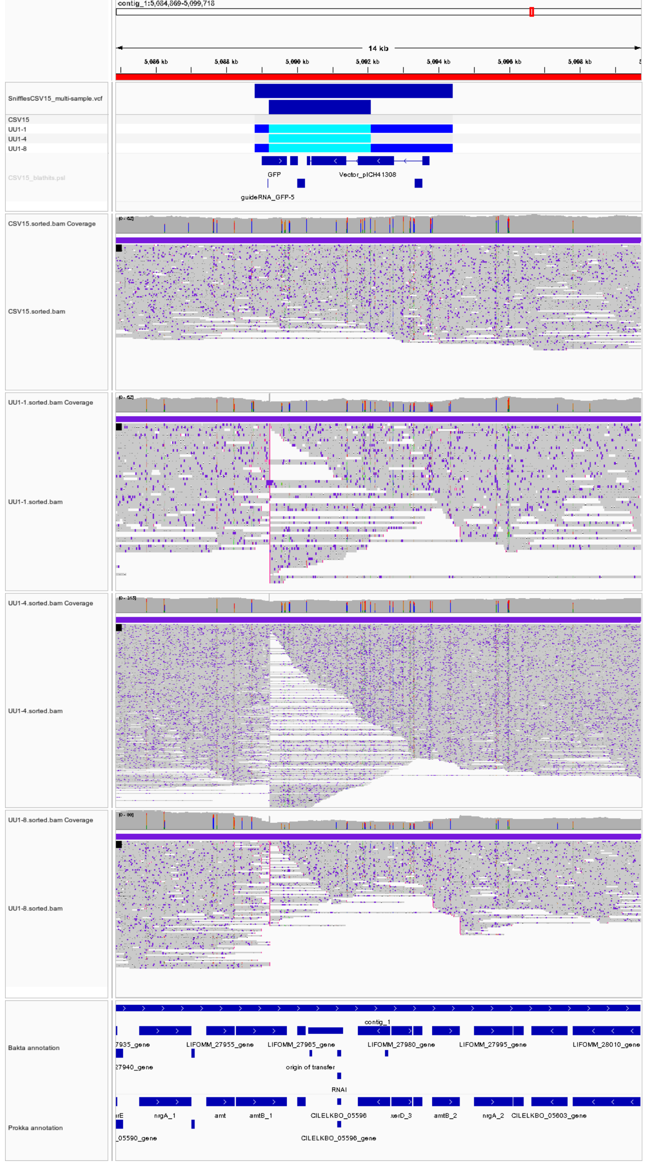

Supplemental Figure S10 Visualization in IGV of the *amt1::gfp* locus for indels 7 (contig_1_5088816_Sniffles2_DEL_AM4) and 8 (contig_1_5089223_Sniffles2_INS_9M4). In UU1-1 and UU1-8, reads were cut sharply at several locations of the plasmid sequences that were inserted when CSVT15 was generated by single cross-over recombination, these are the deletions for indel 7. Penetrance of these events were weak, however. The alignment of the reads is cut sharply and the location of the cargo transposon insertion inside the GFP sequence with 100% penetrance in UU1-1,4 and 8 but not in the parental, this is the true positive insertion from indel 8.

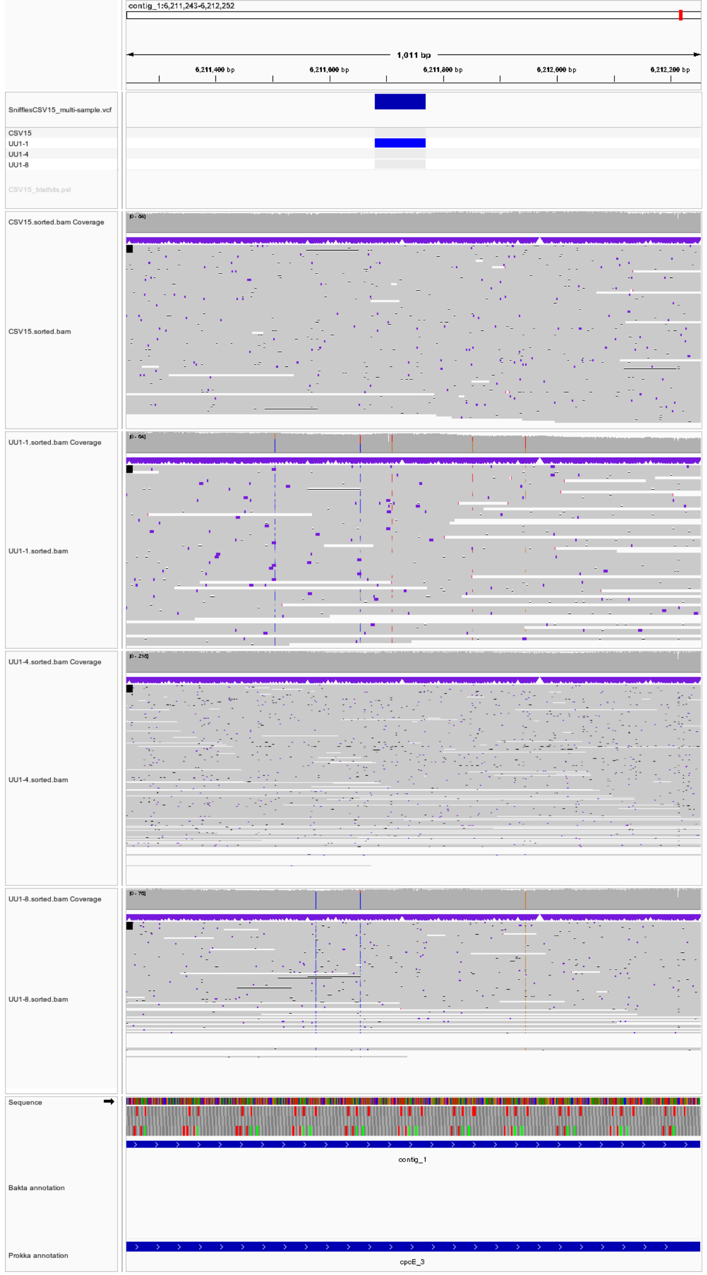

Supplemental Figure S11 Visualization in IGV of the locus for indel 9 (contig_1_6211681_Sniffles2_INS_BM4).
The insertion is short and not supported by many reads.

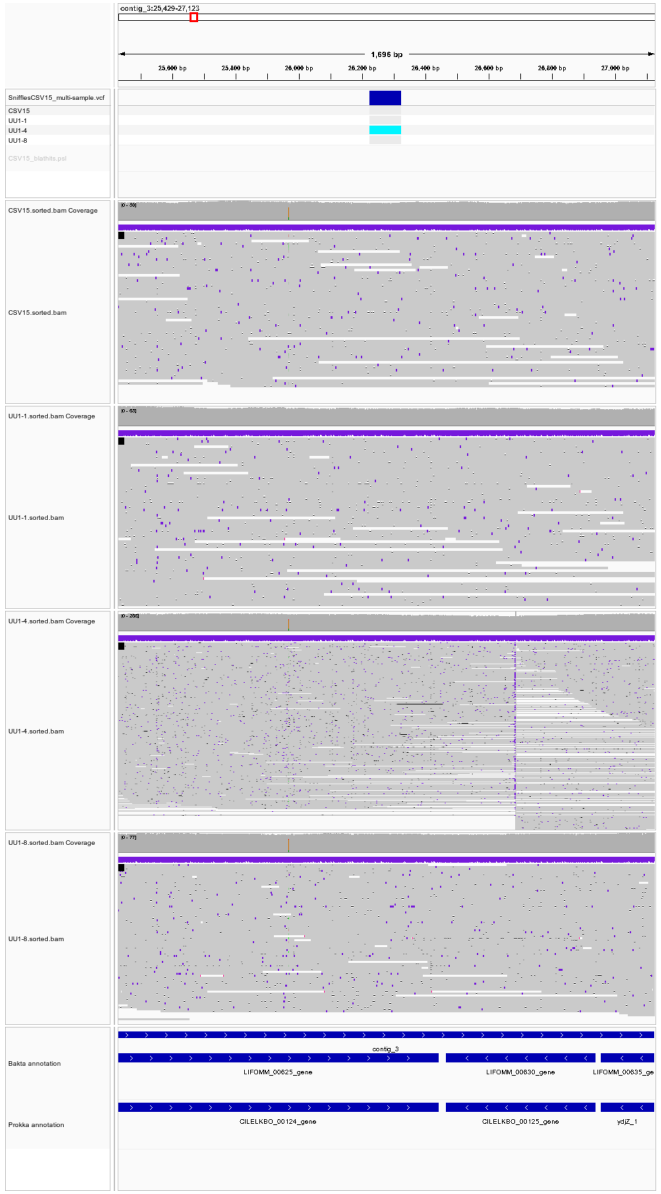

Supplemental Figure S12 Visualization in IGV of the locus for indel 10 (contig_3_26226_Sniffles2_INS_0M1), typical of the indels detected by Sniffles 2 in the plasmid sequences. The indels in plasmids more often than not were located in repetitive sequences encoding transposase.

Plasmid Name	Level	Description
**pAzU0.1**	0	GlnA promoter in the pICH41295 plasmid
**pAzU0.2**	0	TnsB,C,Q sequence in the pICH41308 plasmid
**pAzU0.3**	0	Cas12k sequence in the pICH41308 plasmid
**pAzU0.4**	0	Erythromycin resistance cassette in the pICH41308 plasmid
**pAzU0.5**	0	Spectinomycin resistance cassette in the pICH41308 plasmid
**pAzU1.1**	1	GlnA promoter::TnsB,C,Q::PheA terminator in the pICH47732 plasmid
**pAzU1.2**	1	GlnA promoter::Cas12k::PsaB terminator in the pICH47742 plasmid
**pAzU1.3**	1	J23119 promoter::sgRNA::T7Te terminator in the pICH47751 plasmid
**pAzU1.3.1**	1	J23119 promoter::sgRNA::T7Te terminator in the pICH47751 plasmid. The sgRNA bears GGTT as PAM sequence and hybridizes with the GFP gene
**pAzU1.3.2**	1	J23119 promoter::sgRNA::T7Te terminator in the pICH47751 plasmid. The sgRNA bears GGTT as PAM sequence and hybridizes with the GFP gene in the antisense sequence
**pAzU1.3.3**	1	J23119 promoter::sgRNA::T7Te terminator in the pICH47751 plasmid. The sgRNA bears GTT as PAM sequence and hybridizes with the GFP gene
**pAzU1.3.4**	1	J23119 promoter::sgRNA::T7Te terminator in the pICH47751 plasmid. The sgRNA bears GTT as PAM sequence and hybridizes with the UrtA gene
**pAzU1.3.5**	1	J23119 promoter::sgRNA::T7Te terminator in the pICH47751 plasmid. The sgRNA bears GTT as PAM sequence and hybridizes with the PsbA2 gene
**pAzU1.3.6**	1	J23119 promoter::sgRNA::T7Te terminator in the pICH47751 plasmid. The sgRNA hybridizes with the GFP gene and does not have any PAM sequence
**pAzU1.3.7**	1	J23119 promoter::sgRNA::T7Te terminator in the pICH47751 plasmid. The sgRNA hybridizes with the GFP gene in the antisense sequence and does not have any PAM sequence
**pAzU1.4**	1	LB::RnpB promoter::eYFP::rrnB terminator in the pICH47761 plasmid
**pAzU1.5**	1	cep-oc terminator::Erythromycin resistance::Cpc560 promoter::RB in the pICH47841 plasmid
**pAzU1.6**	1	rnpB promoter::eYFP::rrnB terminator in the pICH47732 plasmid
**pAzU1.7**	1	Cpc560 promoter::Erythromycin resistance::cpc operon in the pICH47742 plasmid
**pAzU1.8**	1	Cpc560 promoter::Spectinomycin resistance::cpc operon in the pICH47742 plasmid
**pAzU1.9**	1	rnpB promoter::eYFP::rrnB terminator in the pICH47761 plasmid
**pAzU1.10**	1	Cpc560 promoter::Erythromycin resistance::cpc operon in the pICH47841 plasmid
**pAzU1.11**	1	Cpc560 promoter::Spectinomycin resistance::cpc operon in the pICH47841 plasmid

Supplemental Table S1 CASTGATE vectors generated in this study. The Level 0 and 1 vectors are presented in Figure 1. Level T are schematically represented and are generally made from combinations of the Level 1 modules. pAzUT.1 until 15, were made with the Level T backbone provided by the CYANOGATE pCAT.000. pAzUT.10 until 15 are designed to test the different sgRNA targeting *gfp-mut2,* either with a conjugative and replicative backbone, or with a replicative backbone only. pAzUT.16 is derived from the pCAT.000 but it lacks coding sequences for replication in cyanobacteria (OriT) which was substituted for ColE1, it is therefore the backbone donor to generate suicide vectors that may be transferred by conjugation, such as for example pAzUT.17, 20, 21 and 22. pAzUT.18 and 19 are designed to target the cargo transposon into expressed genes of wild-type loci from *Anabaena,* the *psbA2* and *urtA*. These vectors are made available in *E.coli* via Addgene. Highlighted in yellow are second-generation vectors used to generate the supplemental data but incompletely tested.

Plasmid Name	Level	Description	Schematic
**pAzUT.1**	T	eYFP, Erythromycin resistance expression cassettes with end linker from the pICH41744 plasmid in the pCAT.334 vector	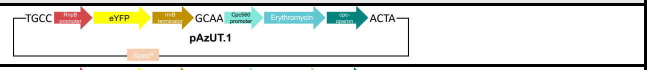
**pAzUT.2**	T	eYFP, Spectinomycin resistance expression cassettes with end linker from the pICH41744 plasmid in the pCAT.334 vector	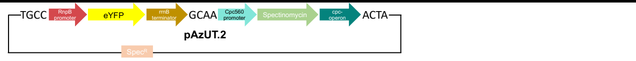
**pAzUT.3**	T	eYFP, Erythromycin resistance expression cassettes with end linker from the pICH41744 plasmid in the pCAT.000 vector	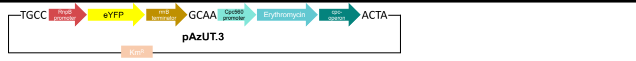
**pAzUT.4**	T	eYFP, Spectinomycin resistance expression cassettes with end linker from the pICH41744 plasmid in the pCAT.000 vector	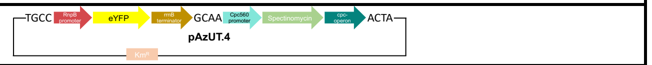
**pAzUT.5**	T	TnsB,C,Q, Cas12k, sgRNA scaffold only, eYFP and Erythromycin expression cassettes with end linker from pICH41800 plasmid in the pCAT.000 vector	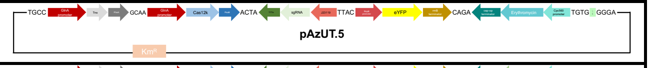
**pAzUT.6**	T	TnsB,C,Q, Cas12k, sgRNA scaffold only, eYFP and Spectinomycin resistance expression cassettes with end linker from the pICH41800 plasmid in the pCAT.000 vector	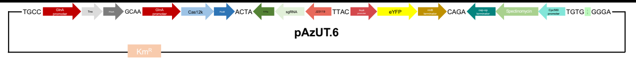
**pAzUT.7**	T	Dummy (from the pICH54011 plasmid), Cas12k, sgRNA scaffold only, eYFP, Erythromycin resistance expression cassettes with end linker from the pICH41800 plasmid in the pCAT.000 vector	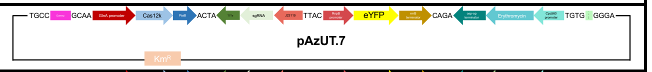
**pAzUT.8**	T	Dummy (from the pICH54011 plasmid), Cas12k, sgRNA scaffold only, eYFP, Spectinomycin resistance expression cassettes with end linker from the pICH41800 plasmid in the pCAT.000 vector	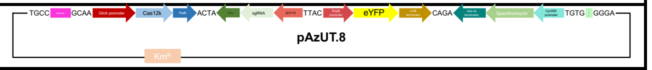
**pAzUT.9**	T	TnsB,C,Q, Cas12k, sgRNA scaffold expression cassettes, RE bordering the transposon cargo right end (RB), eYFP and Erythromycin resistance, LE bordering the transposon cargo left end (LB) with end linker from the pICH41800 plasmid in the pCAT.000 vector	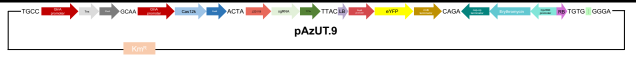
**pAzUT.10**	T	TnsB,C,Q, Cas12k, sgRNA targeting GFP(12), RB, eYFP and Erythromycin resistance expression cassettes, LB with end linker from pICH41800 plasmid in the pCAT.000 vector	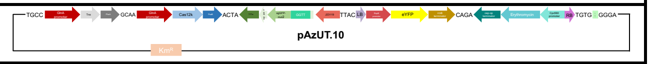
**pAzUT.11**	T	TnsB,C,Q, Cas12k, sgRNA targeting GFP(12), RB, eYFP and Erythromycin resistance expression cassettes, LB with end linker from pICH41800 plasmid in the pCAT.334 vector	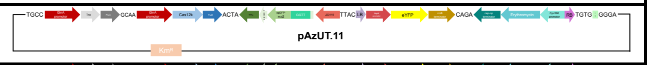
**pAzUT.12**	T	TnsB,C,Q, Cas12k, sgRNA targeting GFP(34), RB, eYFP and Erythromycin resistance expression cassettes, LB with end linker from pICH41800 in the pCAT.000 vector	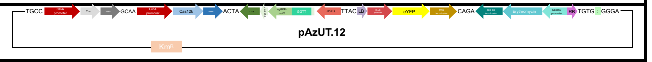
**pAzUT.13**	T	TnsB,C,Q, Cas12k, sgRNA targeting GFP(34), RB, eYFP and Erythromycin resistance expression cassettes, LB with end linker from pICH41800 plasmid in the pCAT.334 vector	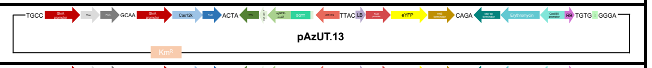
**pAzUT.14**	T	TnsB,C,Q, Cas12k, sgRNA targeting GFP(56), RB, eYFP and Erythromycin resistance expression cassettes, LB with end linker from pICH41800 plasmid in the pCAT.000 vector	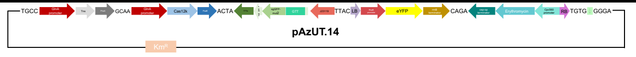
**pAzUT.15**	T	TnsB,C,Q, Cas12k, sgRNA targeting GFP(56), RB, eYFP and Erythromycin resistance expression cassettes, LB with end linker from pICH41800 plasmid in the pCAT.334 vector	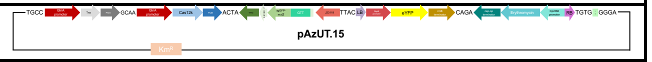
**pAzUT.16**	T	pCAT.000 modified. Replacement of the native OriT with the ColE1 Ori	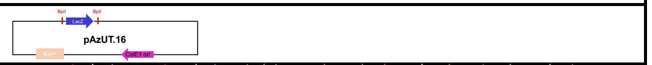
**pAzUT.17**	T	TnsB,C,Q, Cas12k, sgRNA targeting GFP(56), RB, eYFP and Erythromycin resistance expression cassettes, LB with end linker from pICH41800 plasmid in the pAzUT.16 vector	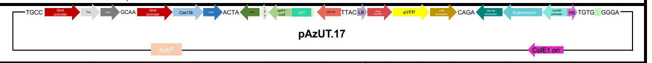
**pAzUT.18**	T	TnsB,C,Q, Cas12k, sgRNA targeting PsbA2, RB, eYFP and Erythromycin resistance expression cassettes, LB with end linker from pICH41800 plasmid in the pCAT.000 vector	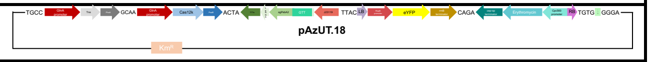
**pAzUT.19**	T	TnsB,C,Q, Cas12k, sgRNA targeting UrtA, RB, eYFP and Erythromycin resistance expression cassettes, LB with end linker from pICH41800 plasmid in the pCAT.000 vector	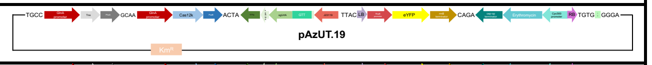
**pAzUT.20**	T	TnsB,C,Q, Cas12k, sgRNA targeting GFP(12), RB, eYFP and Erythromycin resistance expression cassettes, LB with end linker from pICH41800 plasmid in the pAzUT.16 vector	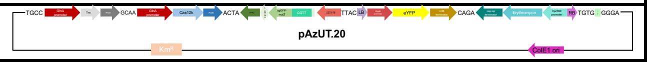
**pAzUT.21**	T	TnsB,C,Q, Cas12k, sgRNA targeting GFP (without PAM sequence), RB, eYFP and Erythromycin resistance expression cassettes, LB with end linker from pICH41800 plasmid in the pAzUT.16 vector	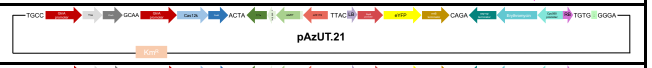
**pAzUT.22**	T	TnsB,C,Q, Cas12k, sgRNA targeting GFP (without PAM sequence and antisense hybridization), RB, eYFP and Erythromycin resistance expression cassettes, LB with end linker from pICH41800 plasmid in the pAzUT.16 vector	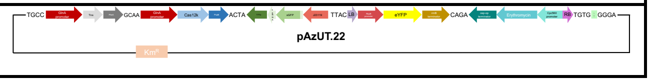

	Strains		
Plasmid (brief description)	Anabaena PCC7120 (WT)	CSVT15 (amt1::gfp)	CSAM137 (sepJ::gfp)
pAzUT.3 (only eYFP and AB cassette) Erythromycin R	✓	✓	✓
pAzUT.4 (only eYFP and AB cassette) Sp/Sm R	✓	✓	✓
pAzUT.5 (- Target, LB and RB)	✓	✓	✓
pAzUT.6 (- Target, LB and RB)	✓		
pAzUT.7 (- Tns and target)	✓	✓	✓
pAzUT.8 (- Tns and target)	✓		
pAzUT.9 (- Target)	✓	✓	✓
pAzUT.10 (gGFP option 1)		✓	✓
pAzUT.12 (gGFP option 2)	✓	✓	✓
pAzUT.14 (gGFP option 3)	✓	✓	✓
pAzUT.17 (Conjugative suicide and gGFP 3)		✓	✓
pAzUT.18 (pCAT.000 gPsbA2)	✓		
pAzUT.19 (pCAT.000 gUrtA)	✓		
pAzUT.20 (Conjugative suicide and gGFP 1)		✓	✓

Supplemental Table S2 CASTGATE vectors transferred to and tested in *Anabaena* wild-type, and the CSVT15 and CSAM137 strains in this study. CSVT15 had been generated by single cross-over recombination so as to generate the *amt1::gfp* fusion (Merino-Puerto et al., 2010) and grew on $BG11_0$ medium without nitrogen. Similarly, CSAM137 had been generated by recombination so as to generate the *sepJ::gfp* fusion (Flores et al., 2007); CSAM137 grew only slowly if at al on the $BG11_0$ medium.

Insertion nr	type	genomic region	sequence ID	length	CSVT15	UU1.1	UU1.4	UU1.8	IGV quality control	annotation
**1**	insertion	chromosome	contig_1_86_Sniffles2_INS_0M4	172	X	X	X	X	noise at contig extremity	transposase
**2**	insertion	chromosome	contig_1_4114_Sniffles2_INS_1M4	8189			X		noise at contig extremity	Trichormus hypothetical protein
**3**	deletion	chromosome	contig_1_2350996_Sniffels2.DEL_4M4	-1467	X	X			present already in CSVT15	IS110 transposase
**4**	deletion	chromosome	contig_1_2444322_Sniffles2_DEL_5M4	-693	X	X	X	X	assembly issue in CSVT15 reference	Histidine kinase response regulator
**5**	insertion	chromosome	contig_1_4325042_Sniffles2_INS_6M4	77		X			false positive	no good hit
**6**	recombination	chromosome	contig_1_4886060_Sniffles2_BND_8M4	NA			X		true in UU1.4	NA
**7**	deletion	chromosome	contig_1_5088816_Sniffles2_DEL_AM4	-5613		X		X	true in UU1.1 and UU1.8	homologous recombination of GFP plasmid
**8**	insertion	chromosome	contig_1_5089223_Sniffles2_INS_9M4	2893		X	X	X	positive	CAST insertion
**9**	insertion	chromosome	contig_1_6211681_Sniffles2_INS_BM4	89		X			false positive	HEAT repeat domain containing protein / glycosyltransferase
**10**	insertion	plasmid	contig_3_26226_Sniffles2_INS_0M1	99			X		false positive	IS5 transposase
**11**	insertion	plasmid	contig_4_56839_Sniffles2_INS_0M0	1658		X			positive	ISNCY transposase
**12**	insertion	plasmid	contig_4_82848_Sniffles2_INS_1M0	905		X			positive	no good hit
**13**	insertion	plasmid	contig_5_49595_Sniffles2_INS_0M2	205		X			positive	-
**14**	insertion	plasmid	contig_5_139017_Sniffles2_INS_2M2	1672		X			positive	NIES-23 transposase
**15**	recombination	plasmid	contig_5_148784_Sniffels2_BND_5M2	NA			X		positive	transposase
**16**	recombination	plasmid	contig_5_151176_Sniffels2_BND_5M2	NA			X		positive	same transposase as above
**17**	insertion	plasmid	contig_5_174398_Sniffles2_INS_3M2	221			X		positive	ISNCY transposase
**18**	insertion	plasmid	contig_5_193393_Sniffles2_INS_4M2	1666				X	positive	
**19**	insertion	plasmid	contig_5_260548_Sniffles2_INS_7M2	1671		X			positive	ISNCY transposase
**20**	insertion	plasmid	contig_5_266694_Sniffles2_INS_8M2	1467		X			positive	ISNCY transposase
**21**	recombination	plasmid	contig_5_267538_Sniffles2_BND_9M2	NA				X	positive	
**22**	insertion	plasmid	contig_5_399380_Sniffles2_INS_AM2	1670			X		positive	ISNCY transposase

Supplemental Table S3 All the indels detected by Sniffles 2 comparing genome assemblies from the parental strain with those from clones obtained after RNA-guided transposition in the *amt1::gfp* locus. Sequence identity was specified starting with the contig in which it was encoded. Contig_1 encoded the chromosome. X identifies detection of the indel in either the parental (CSVT15) or the strains with the RNA-guided insertion of the cargo transposon in the *amt1::gfp* locus (UU1.1, UU1.4 and UU1.8). Contig_2- to contig_5 encompassed the plasmids (plasmid assemblies seemed more fluid, possibly because of assembly issues). The analyses were highly dependent on the quality of the assemblies, hence the quality control in IGV to visualize the sequence reads aligned to the parental CSVT15 genome assembly (IGV quality control). We provide the IGV-visualizations to substantiate conclusions for all the 9 indels detected in the chromosome in Supplemental Figures S5-S11, we also provide the example for a plasmid indel 10 in Supplemental Figure S12.

Name	Sequence	PCR-assay/cloning step
**Amt1-1**	GAGTCACCAGAGAAGAAGAATTGGA	Fig.2 PCR1 and PCR3
**Ery-5**	CCTGATGAATGAGGGTAACACG	Fig. 1 and Fig.2 PCR2
**eYFP-5**	GCAGATGAACTTCAGGGTCAG	Fig.1 and Fig.2 PCR1
**GFP-3**	CCATGCCATGTGTAATCCCAG	Fig.1 and Fig. 2 PCR2
**SepJ-1**	CCCAAATATTCGGAGTTTTATTCGTCA	Fig.1 PCR1 and PCR3
**pCAT.000-10**	GATACCTTGTGCGGCTATGT	Fig.1 and Fig.2 PCR4
**pCAT.000-11**	GTTTGGTTGATGCGAGTGATTT	
**Ery1-F**	TTGAAGACAAAATGAATAAAAATATTAAATACTCTC	Amplification of erythromycin resistance gene CDS from pC0.029
**Ery2-R**	TTGAAGACAAAAGCTTATTTCCGCCCATTAAACAAC	
**ProGlnA_1**	TTGAAGACTTGGAGCGCATTCCTTCTTCTC	Amplification of the P_{GlnA} for insertion into Level 0 Promoter + 5 UTR
**ProGlnA_2**	TTGAAGACAAATGGTGTTACTCCTTCTCTGCCA	Amplification of the P_{GlnA} for insertion into Level 0 Promoter + 5 UTR
**ProGlnA_3**	TTGAAGACAACATTTGTTACTCCTTCTCTGCCA	Pair with ProGlnA_1 to clone Pro+5U with ATG
**Spe1-F**	TTGAAGACAAAATGCGCGAAGCGGTTATTG	Amplification of spectinomycin resistance gene CDS from pC0.028
**Spe2-R**	TTGAAGACAAAAGCTTACTTGCCGACCACTTTTG	
**LB-Bsa-F1**	TTGGTCTCAAGCGAGGCGTAGTGTACAGTGAC	Amplification of Left End from pDONOR (Strecker et al., 2019) for the Level 1 construct
**LB-Bsa-R2**	TTGGTCTCAGGAGTCAGTAACTATTAAACTTAGGGGTGGG	
**LB-Bsa-F3**	TTGGTCTCAGGAGAGGCGTAGTGTACAGTG	Amplification of Left End from pDONOR (Strecker et al., 2019) to fuse to the Promoter-eYFP-Terminator cassette
**LB-Bsa-R4**	TTGGTCTCAAGTATCAGTAACTATTAAACTTAGGGGTG	
**RB-Bsa-F1**	TTGGTCTCAAGCGAAGGCGACAGTCAATTTGTC	Amplification of Right End from pDONOR (Strecker et al., 2019) for the Level 1 construct
**RB-Bsa-R2**	TTGGTCTCAGGAGCTACGTCTCTACGTGTACAG	
**RB-Bsa-F4**	TTGGTCTCATACTAAGGCGACAGTCAATTTGTC	Amplification of Left End from pDONOR (Strecker et al., 2019) to fuse to a Promoter-EryR-Terminator cassette. Use with RB-Bsa-R2
**gGFP-1 (F1)**	TTGCTCTTCCAAAGGTTATGTACAGGAAAGAACTATATTTTTCAAAGGCTGGAAGAGCAA	gRNA of GFP-Mut2 using GGTT as PAM sequence and restriction enzyme LguI. R1 is a complement strand of F1. Used for pAzUT.10
**gGFP-2 (R1)**	TTGCTCTTCCAGCCTTTGAAAAATATAGTTCTTTCCTGTACATAACCTTTGGAAGAGCAA	
**gGFP-3 (F2)**	TTGCTCTTCCAAAGGTTGTCTGGTAAAAGGACAGGGCCATCGCCAATGCTGGAAGAGCAA	gRNA targeting GFP-Mut2 using GGTT as PAM sequence in complement strand, and restriction enzyme LguI. R2 is a complement strand of F2. Used for pAzUT.12
**gGFP-4 (R2)**	TTGCTCTTCCAGCATTGGCGATGGCCCTGTCCTTTTACCAGACAACCTTTGGAAGAGCAA	
**gGFP-5 (F3)**	TTGCTCTTCCAAAGTTCCATGGCCAACACTTGTCACTACTTTCGCGGCTGGAAGAGCAA	gRNA of GFP-Mut2 using GTT as PAM sequence and restriction enzyme LguI. R3 is a complement strand of F3. Used for pAzUT.14
**gGFP-6 (R3)**	TTGCTCTTCCAGCCGCGAAAGTAGTGACAAGTGTTGGCCATGGAACTTTGGAAGAGCAA	

Supplemental Table S4 Primers used for PCR assays and key cloning steps in this study.
